# Perihematomal Edema and Functional Outcome After Intracerebral Hemorrhage: A Meta-Analysis of Individual Participant Data

**DOI:** 10.1161/STROKEAHA.125.053991

**Published:** 2026-03-04

**Authors:** Neshika Samarasekera, Sharon Tuck, Xia Wang, Craig S. Anderson, Alireza Shirazian, Bastian Volbers, Hagen B. Huttner, Sarah Marchina, Casey Norton, Magdy Selim, Kevin Sheth, Guido J. Falcone, Pedro Castro, Grant Mair, Tom J. Moullaali, Mark A. Rodrigues, Adrian Parry-Jones, Nikola Sprigg, Floris H.B.M. Schreuder, Thorsten Steiner, Ken S. Butcher, Daniel Hanley, Wendy Ziai, Andrew Demchuk, David Rodriguez-Luna, Ramon Iglesias Rey, Pablo Hervella, Atte Meretoja, Aaron M. Gusdon, Christopher J. Weir, Rustam Al-Shahi Salman

**Affiliations:** Institute for Neuroscience and Cardiovascular Research (N. Samarasekera, G.M., T.J.M., M.A.R., R.A.-S.S.), The University of Edinburgh, United Kingdom.; Edinburgh Clinical Trials Unit, Usher Institute (S.T., C.J.W., R.A.-S.S.), The University of Edinburgh, United Kingdom.; The George Institute for Global Health, Faculty of Medicine (X.W., C.S.A., R.A.-S.S.), University of New South Wales, Sydney, Australia.; School of Clinical Medicine (K.S.B.), University of New South Wales, Sydney, Australia.; Department of Neurology, University of Chicago Medical Center, IL (A.S.).; Department of Neurology, University Hospital Erlangen, Friedrich-Alexander-Universität Erlangen-Nürnbergt Erlangen-Nrnberg, Germany (B.V., H.B.H.).; Department of Neurology, Beth Israel Deaconess Medical Center, Stroke Division, Boston, MA (S.M., C.N., M.S.).; Department of Neurology, Yale University School of Medicine, New Haven, CT (K.S., G.J.F.).; Department of Clinical Neurosciences and Mental Health, Faculty of Medicine, University of Porto, Portugal (P.C.).; Department of Neurology, Centro Hospitalar Universitário de São Joãorio de So Joo, Porto, Portugal (P.C.).; Geoffrey Jefferson Brain Research Centre, Manchester Academic Health Science Centre, Northern Care Alliance NHS Foundation Trust & University of Manchester, United Kingdom (A.P.-J.).; Stroke Trials Unit, Mental Health and Clinical Neuroscience, University of Nottingham, Queens Medical Centre Hospital Campus, United Kingdom (N. Sprigg).; Department of Neurology, Donders Institute for Brain, Cognition and Behaviour, Radboud University Medical Centre, Nijmegen, the Netherlands (F.H.B.M.S.).; Department of Neurology, Klinikum Frankfurt Höchst, Germany (T.S.).; Division of Neurology, University of Alberta, Edmonton, Canada (K.S.B.).; Division of Brain Injury Outcomes, Johns Hopkins University, Baltimore, MD (D.H., W.Z.).; Department of Clinical Neurosciences and Radiology, Hotchkiss Brain Institute, University of Calgary, Alberta, Canada (A.D.).; Department of Neurology, Vall d’Hebron University Hospital, Barcelona, Spain (D.R.-L.).; Neuroimaging and Biotechnology Laboratory (NOBEL), Clinical Neurosciences Research Laboratory, Health Research Institute of Santiago de Compostela, Spain (R.I.R., P.H.).; Department of Neurology, Helsinki University Hospital, Finland (A.M.).; Department of Neurosurgery, University of Texas Health Science Center, McGovern School of Medicine, Houston (A.M.G.).

**Keywords:** biomarkers, brain, cerebral hemorrhage, edema, tomography

## Abstract

**BACKGROUND:**

Perihematomal edema (PHE) after intracerebral hemorrhage (ICH) is a biomarker of secondary brain injury. We aimed to determine the direction, strength, and temporality of the association between PHE and functional outcome after ICH onset.

**METHODS:**

We did a systematic review to identify cohort studies or trials that used brain computed tomography (CT) imaging to diagnose ICH, and measured functional outcome. We sought individual participant data if they had a diagnostic CT within 72 hours, a repeat CT within 14 days of the diagnostic scan, and were not treated with surgery or therapy that could affect PHE. We did a 2-stage individual participant data meta-analysis. The primary analysis was the association between the change in absolute PHE volume between the diagnostic CT and repeat CT and the primary outcome of death or dependence (modified Rankin Scale score, 3–6) at 90±14 days after ICH onset. We quantified the association between change in absolute PHE volume at 2 repeat CT time points (24±12 and 72±12 hours) and outcome, both unadjusted and adjusted for age, sex, ICH volume on the diagnostic CT, and intraventricular extension using multivariable logistic regression.

**RESULTS:**

From 12 969 studies, 38 were eligible, of which 12 studies (with 1 unpublished cohort and the VISTA [Virtual International Stroke Trials Archive]-ICH databank) provided data. We included 1523 participants, of whom 1347 participants (516 [38%] participants female; median age, 66 [interquartile range, 55–75] years) had repeat CT at 24±12 hours, and 495 (195 [39%] participants female; median age, 66 [interquartile range, 55–74] years) had repeat CT at 72±12 hours. 319 participants contributed to both analyses. Death or dependence was associated with absolute PHE growth both in the first 24±12 hours (unadjusted odds ratio, 1.04 per mL increase [95% CI, 1.01–1.06]; *P*<0.01; adjusted odds ratio, 1.04 per mL increase [95% CI, 1.01–1.06]; *P*<0.01) and in the first 72±12 hours (unadjusted odds ratio, 1.03 per mL [95% CI, 1.01–1.04]; *P*<0.01; adjusted odds ratio, 1.02 [95% CI, 1.01–1.04] per 1 mL increase; *P*<0.01).

**CONCLUSIONS:**

PHE growth within 24 and 72 hours of ICH onset is independently associated with death or dependence after ICH.

Spontaneous intracerebral hemorrhage (ICH) is a devastating form of stroke whose global burden has increased over the last 30 years.^1^ One promising treatment target^2^ is secondary injury, which evolves soon after onset and consists of several processes, including clot retraction, vasogenic edema formation, red cell lysis, and inflammation, and for which perihematomal edema (PHE) is an imaging biomarker.^3–5^

Two systematic reviews using aggregate data^6,7^ have shown an association between PHE and functional outcome after ICH, although these reviews were limited by heterogeneity in the assessment of PHE and functional outcome. Growth of PHE after ICH onset (rather than PHE measured *at* a specific time point after onset) may be more strongly associated with outcome.^7^ However, analyses have been post hoc, combined studies that assessed PHE growth during different time periods after onset^6^ and have not adjusted for other variables that are known to affect ICH outcome.^6,7^

There is increasing awareness that the concept of time is brain applies not only to ischemic stroke but also to ICH,^8^ which led to the Code ICH^9^ paradigm for improving acute ICH care. Knowing that PHE is independently associated with functional outcome, how PHE evolves after ICH onset, and determining when it is associated with outcome would inform the design of randomized controlled trials of interventions for PHE.

We aimed to do an individual participant data meta-analysis to (1) describe the trajectory of PHE growth after ICH onset, (2) confirm whether PHE is independently associated with functional outcome, and (3) determine the strength of this association over time after ICH onset.

## Methods

### Protocol and Registration

We registered the protocol (page 15 in the Supplemental Material) with PROSPERO (https://www.crd.york.ac.uk/PROSPERO/view/CRD42021253263) and agreed on a prespecified statistical analysis plan (unpublished, finalized on July 6, 2022; page 25 in the Supplemental Material). We used PRISMA-IPD guidelines for reporting (Preferred Reporting Items for Systematic reviews and Meta-Analyses-Individual participant data; Supplemental Material).

### Data Availability

Contributing cohorts shared data on the condition that the data recipient (The University of Edinburgh) shall not sublicense, transfer, disclose, or otherwise make available the data in whole or part to any third party except with specific prior written consent from the data provider. Written requests will be assessed by representatives of the contributing cohorts and the corresponding author, and a decision will be made about the appropriateness of the reuse of data. A data sharing agreement will be put in place before any data are shared. Individual cohort data sets may be shared according to the terms described by each cohort.

### Study Eligibility Criteria and Methods of Identifying Studies

We did a systematic review^6^ to identify studies that used brain computed tomography (CT) to measure PHE after ICH onset and would share participant-level data for this meta-analysis.

We identified potentially eligible cohorts by searching OVID Medline (1950-; RRID:SCR_002185) and Embase (1980-; RRID:SCR_001650), ClinicalTrials.gov, ISRCTN Registry, and Cochrane Reviews|Cochrane Library (RRID:SCR_013000) to November 17, 2021, by using a search strategy comprising terms for ICH and PHE,^6^ hand searching the bibliographies of relevant studies, and contacting authors of collaborating studies. We included the largest single report of any observational cohort or randomized trial involving ≥10 participants with ICH that measured PHE and functional outcome. If an intervention in a randomized trial could have affected PHE (eg, steroids, mannitol, or hypertonic saline), we included the control arm only. We included studies irrespective of the language of publication, but excluded conference abstracts. We excluded studies solely using magnetic resonance imaging to assess PHE because CT is the most frequently used imaging modality for participants with ICH, is reliable for the semiautomated assessment of PHE,^10^ and we wished to standardize PHE assessment. We included eligible participants from the control arm of randomized trials in the VISTA (Virtual International Stroke Trials Archive) for ICH (https://www.virtualtrialsarchives.org/vista-ich/) databank.

### Participant Eligibility Criteria

We included participants from these studies if they: were male or female adults (age ≥18 years); had nontraumatic ICH that was probably due to cerebral small vessel disease (not known to be secondary to a structural cause); had a first (diagnostic) CT within 72 hours of ICH symptom onset; and had a repeat CT at least once within 14 days of the diagnostic CT to enable assessment of PHE growth.

We excluded participants treated with any form of neurosurgical intervention for ICH, and participants where the time from ICH onset to diagnostic CT was unknown.

We emailed an invitation to collaborate to the corresponding authors of eligible studies, followed by 1 reminder. We included studies if the corresponding author confirmed eligibility, supplied a data dictionary with their data explaining the variables shared, and provided participant-level data on eligibility criteria and baseline characteristics, including timing of diagnostic and repeat CTs, ICH and PHE volumes on each CT, and timing and measurement of functional outcome.

Research ethics committees overseeing the use of patients’ data had approved the collaborating studies. Cohorts shared only anonymized data, so individual consent was not required, but we obtained approval from the Edinburgh Medical School Research Ethics Committee (21-EMREC-019).

### Data Collection Including Assessment of Risk of Bias

We collected study-level characteristics and participant-level characteristics (Supplemental Material). We used the QUIPS tool to assess the risk of bias of studies that supplied data.^11^ We rated studies that we included in the meta-analysis and studies that supplied data but were excluded, and gave a study an overall low risk of bias if the most important domains, which we selected a priori, had a low risk of bias. We checked data completeness and consistency within each cohort and resolved any queries directly with the corresponding authors. We standardized the format, coding, and units of measurement of variables to maximize the number available for analysis in all studies. We combined data sets from collaborating studies into a new master data set, which included a variable to indicate the original study. We did not use or request aggregate data from cohorts that did not share participant-level data.

Because methods of measuring PHE volumes may differ and PHE volume is closely associated with ICH volume, we calculated the ratio of ICH-to-PHE volume for each study. We used this as a measure of the consistency of PHE volume measurement between cohorts for a given time point after ICH onset and hypothesized that ratios would be comparable if methods of measurement were similar. Blinding did not apply in this individual participant data meta-analysis in which PHE had been measured by collaborating cohorts.

### Primary and Secondary Analyses

We used the modified Rankin Scale score 3 to 6 at 90 (±14) days as the measure of our primary outcome of death or dependence, because this was the most frequently used outcome measure in the participating studies.

We prespecified that the primary analysis would be the association between the change in PHE volume between the diagnostic CT and a repeat CT in the first 14 days after the diagnostic CT and death or dependence. We did not prespecify a time point for the repeat CT after the diagnostic CT because we wanted to select the time point (±12 hours) after the diagnostic CT when we had the most repeat CTs to maximize data availability. We prespecified absolute volume as our PHE measure because this is the most frequently used measure of PHE.^6,7^

The prespecified secondary analyses were (1) the association between PHE and functional outcome at different time points, between which change in PHE volume was measured, and (2) the association between PHE volume at a time point (±12 hours) in the first 2 weeks after the diagnostic CT and death or dependence using the same time point as the primary analysis. In a post hoc analysis, we plotted the value of absolute PHE volume at each time point.

### Synthesis of Results

We prespecified unadjusted and adjusted models for the primary and secondary analyses. We used a 2-stage random effects logistic regression meta-analysis with restricted maximum likelihood estimation and a random intercept and coefficient for each study included in the model. As the included studies were large, we used a 2-stage approach to enable the use of restricted maximum likelihood estimation. This reduces the downward bias in between-study variance estimates, which is not available under the single-stage approach. In stage 1, a logistic regression model was fitted to the data for each study to obtain a log-odds ratio (OR) estimate and SE for the association between the change in absolute PHE volume and death or dependence. At stage 2, these study-specific estimates were combined in a random effects meta-analysis, including a Hartung-Knapp^12^ adjustment to control for the uncertainty in between-study variance estimates by inflating the width of the CI. Random effects modeling allowed for the true association to be different for each study, with t^2^ quantifying the heterogeneity between studies. In a prespecified sensitivity analysis, we compared the random effects model with the common effects (fixed-effects) model.

#### Model Building and Covariate Selection

In the multivariable-adjusted modeling, we sought to prespecify clinical variables from the ICH score^13^ on the basis of their known association with outcome and clinical relevance. Of these prespecified variables, we selected variables according to their completeness and availability at the time of diagnosis in the available cohorts, and the extent to which their selection maximized the total sample size available for multivariable analyses. We sought to include radiological variables (ICH volume, location, and time from onset to baseline imaging) in the multivariable model based on the significance of their univariate association with outcome and the completeness of data. To minimize any bias in the model SE estimates, we did not include variables such as ICH growth, which are known to be collinear with the absolute change in PHE volume.

#### Model Validation

At stage 1, we assessed model fit using the Akaike and Bayesian information criteria. We internally validated the model using bootstrap validation, which, by resampling from the full original data set, allows all of the available data to contribute to model development and permits optimism-adjusted values of model discrimination (C statistic; Brier score) to be calculated.

If there was missing data for a particular variable, those records were removed from any statistical analysis relating to that variable, unless otherwise specified.

There is no universally accepted method for determining sample size in multivariable regression, but we used the maximum sample size available for multivariable analyses. We did not use a power calculation.

We did analyses using R (version 4.2.2, R Core Team, 2023).^14^

## Results

We screened 12 968 articles identified by database searching and identified 1 additional study^15^ and a further unpublished cohort from Sheth et al through contact with researchers. We assessed 159 full-text articles for eligibility. We excluded 22 studies which included participants in a cohort published elsewhere,^16–37^ 19 review articles,^38–56^ 16 studies which had measured PHE on the diagnostic scan only,^57–72^ 13 studies which did not report an outcome measure,^73–85^ 12 studies which assessed PHE using magnetic resonance imaging,^86–97^ 12 studies in which some/all participants had received an intervention which might affect PHE,^98–109^ 11 participants in which some/all had surgery,^110–120^ 8 in which brain edema or cerebral blood flow was measured rather than PHE,^121–128^ 6 which included ICH with ischemic stroke or other intracranial hemorrhage,^129–134^ and 2 studies which had <10 participants.^135,136^ These differ from the systematic review,^6^ which (unlike this individual participant data meta-analysis) excluded studies that assessed PHE and a functional outcome in a single cohort but did not report an association between them or assessed functional outcome using alternatives to the modified Rankin Scale.

**Figure 1.**
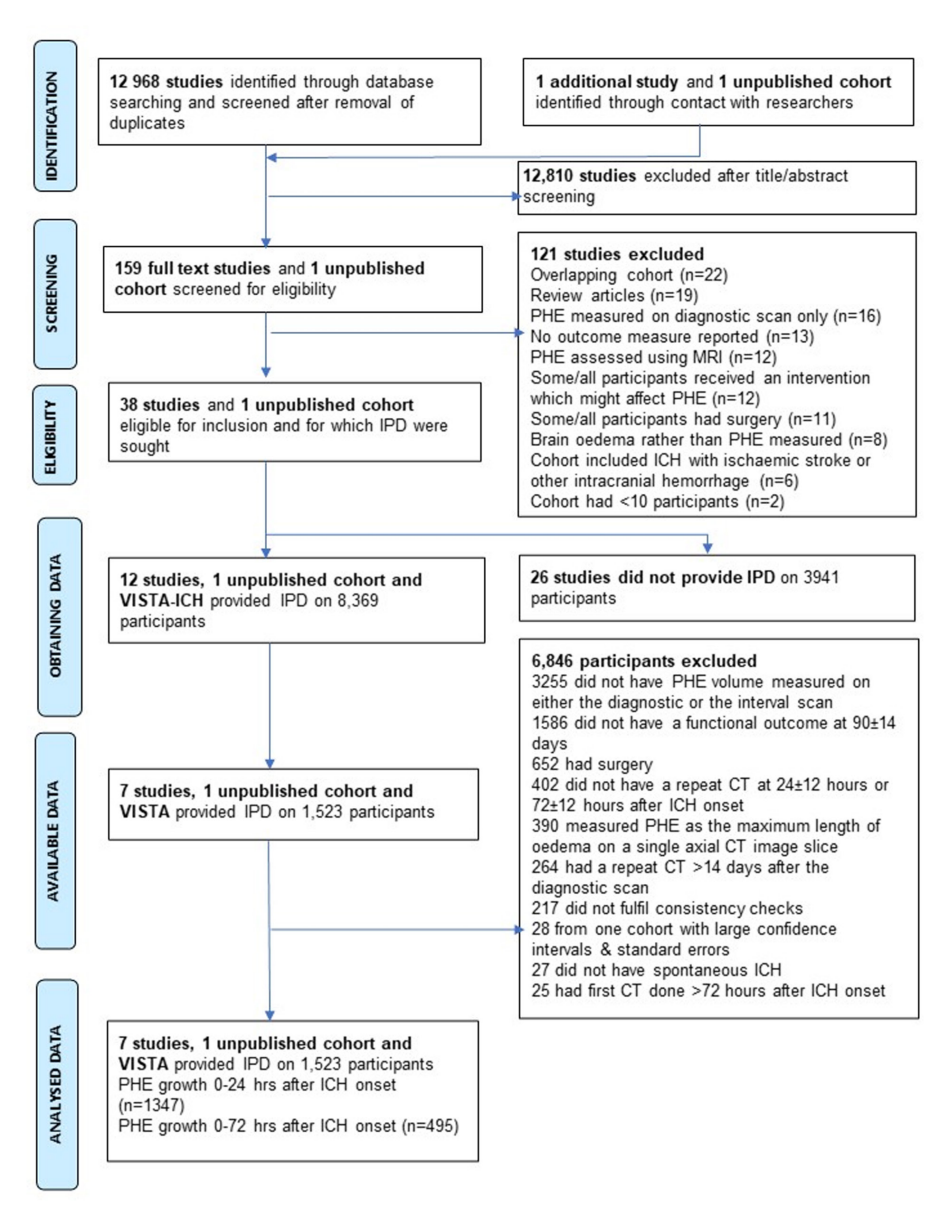
**PRISMA flowchart.** CT indicates computed tomography; ICH, intracerebral hemorrhage; IPD, individual participant data; MRI, magnetic resonance imaging; PHE, perihematomal edema; PRISMA, Preferred Reporting Items for Systematic Reviews and Meta-Analyses; and VISTA, Virtual International Stroke Trials Archive.

We invited 38 eligible studies to share data. 26 eligible studies did not respond to the invitation (Table S1).^137–162^ Twelve studies (32%), the VISTA databank, and 1 unpublished cohort by Sheth et al, shared individual participant data on 8369 participants (Figure 1). We then excluded 6 studies which shared data for the following reasons: 1 measured PHE on their repeat CT as the maximum length of PHE on the axial CT image slice with most edema visible,^163^ 1 did not measure PHE volume on the diagnostic scan,^164^ 2 did not assess functional outcome using the modified Rankin Scale score at 90 days,^15,165^ which was the most commonly used time point for assessing outcome, one study did not pass data consistency checks,^166^ and one, which due to a small number of eligible participants, produced large CIs and SEs.^167^ The remaining studies (Table S2) contributed 1523 participants from 9 cohorts: 3 randomized trials (the control and intervention arms of the first 2 intensive blood pressure reduction in acute cerebral hemorrhage trials [INTERACT 1 (Intensive Blood Pressure Reduction in Acute Cerebral Hemorrhage Trial) and INTERACT 2 (The Second Intensive Blood Pressure Reduction in Acute Cerebral Hemorrhage Trial)] comprising participants predominantly in Asia,^168^ the control arm of the i-DEF trial [Deferoxamine Mesylate in Patients With Intracerebral Hemorrhage], from United States^169^), 4 published observational cohorts (from United States,^170^ United Kingdom,^171^ Germany,^172^ and Spain^173^), 1 unpublished cohort from United States (Sheth et al), and the VISTA databank.

We identified 2 groups for analysis: 1347 participants with repeat brain CT at 24±12 hours (Table; Table S3) and 495 with repeat brain CT at 72±12 hours (Tables S4 and S5); 319 participants contributed to both analyses. All published cohorts except one, which used the ABC/2 formula,^170^ used semiautomated planimetric methods to measure ICH and PHE volumes (Table S2). We found that each published cohort contributing data had a moderate risk of bias for the study participation domain because they were retrospective (including retrospective analyses of prospective cohorts)^34,168,170,173^ or susceptible to selection bias,^169,171^ and 2 studies had a moderate risk of bias in functional outcome measurement because outcome was assessed by review of medical records^170^ or the method of ascertainment was not reported (Tables S6 and S7).^173^ The participant characteristics of those included were similar to those of those excluded (Table S8).

**Table.**
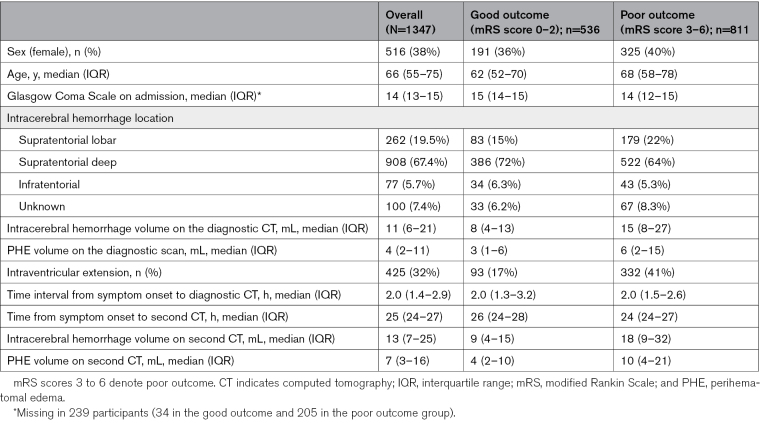
Characteristics of Participants by Functional Outcome Assessed by mRS Score at 90±14 Days

Of 1347 participants with repeat CT at 24±12 hours (Table), 516 participants (38%) were female, and the median age was 66 years (interquartile range [IQR], 55–75 years). ICH locations in 1347 participants were supratentorial deep (n=908, 67%), lobar (n=262, 19%), infratentorial (n=77, 6%), and unknown (n=100, 7%). VISTA-ICH did not provide an ICH location, and 94 (94%) of the participants with an unknown ICH location were from VISTA-ICH. Another cohort did not provide the admission Glasgow coma scale score.^170^

The median time from symptom onset to diagnostic CT was 2 hours (IQR, 1·4–2·9 hours). On the diagnostic CT, median ICH volume was 11 mL (IQR, 6–22 mL) and median absolute PHE volume was 4 mL (IQR, 2–11 mL), which increased to 7 mL (IQR, 3–16 mL) on the 24±12 hour repeat CT. The median ratio of ICH: PHE volume on CTs at 24±12 hours after ICH onset was 1.8 mL (IQR, 0.9–3.5 mL), and only 1 cohort^168^ had a higher ratio (3.3 mL; IQR, 2.1–5.6 mL) in comparison to the rest (Table S9).

Of 495 participants with repeat CT at 72 ±12 hours (page 18 in the Supplemental Material), 195 participants (39%) were female, and the median age was 66 years (IQR, 55–74 years). ICH locations were supratentorial deep (n=287, 58%), lobar (n=78, 16%), infratentorial (n=31, 6.3%), and unknown (n=99, 20%).

**Figure 2.**
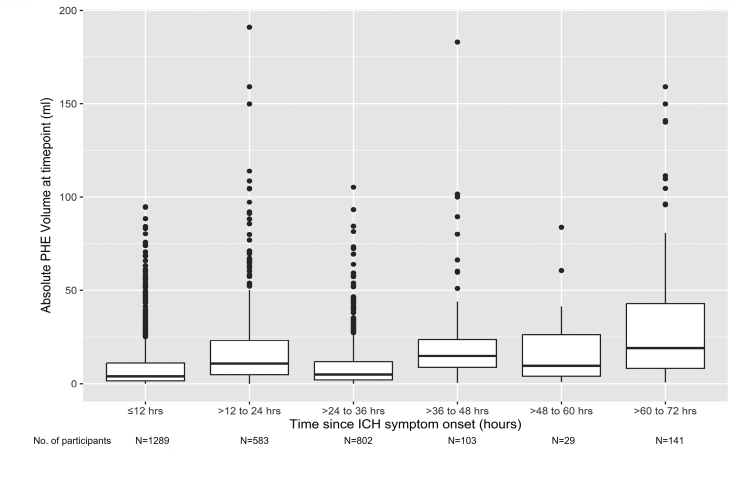
**Box and whisker plot of absolute perihematomal edema PHE volumes in milliliters (mL) in 12 hourly intervals over the first 72 hours after intracerebral hemorrhage (ICH) onset.** Data at each time point are based on the subset of participants in whom computed tomography was performed at that time point.

The median absolute PHE volume was 4.0 mL (IQR, 1.6–11.0 mL) within 12 hours of ICH onset, 14.8 mL (IQR, 8.8–23.7 mL) between 36 and 48 hours, and 19.1 mL (IQR, 8.2–43.0 mL) between 60 and 72 hours of ICH onset (Figure 2).

**Figure 3.**
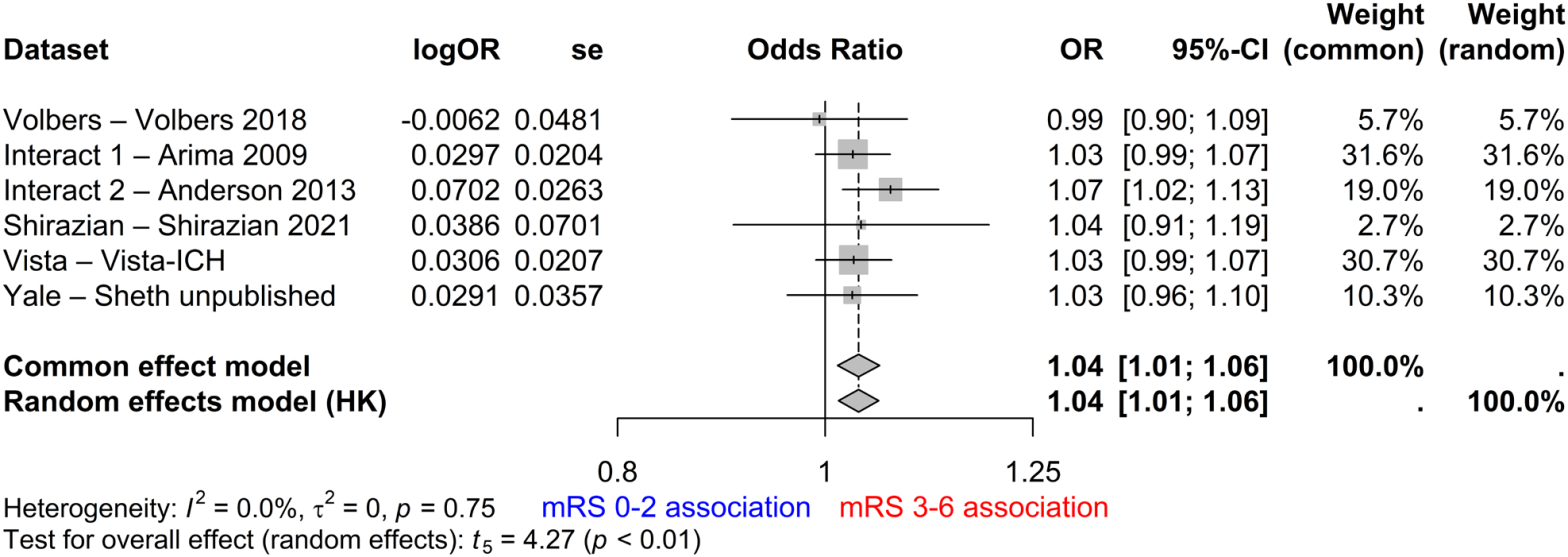
**Forest plot of the adjusted logistic regression model of the association between the change in absolute perihematomal edema volume between the diagnostic computed tomography (CT) and repeat CT at 24±12 hours after intracerebral hemorrhage (ICH) onset and death or dependence at 90 days (N=1347; after adjustment for age, sex, ICH volume, and intraventricular extension).** HK indicates Hartung-Knapp adjustment; mRS, modified Rankin Scale; OR, odds ratio; and VISTA, Virtual International Stroke Trials Archive.

For the primary analysis, using an unadjusted random effects logistic regression model, growth in absolute PHE volume between the diagnostic CT and a repeat CT 24±12 hours after onset was associated with death or dependence (OR, 1.04 per mL [95% CI, 1.01–1.06]; I^2^=25%) with a fixed-effects model showing similar results (OR, 1.04 per mL [95% CI, 1.02–1.05]; Figure S1). These results were unchanged after adjustment for age, sex, ICH volume, and intraventricular extension in both random and fixed-effects models (OR, 1.04 per mL [95% CI, 1.01–1.06]; I^2^=0%; Figure 3).

**Figure 4.**
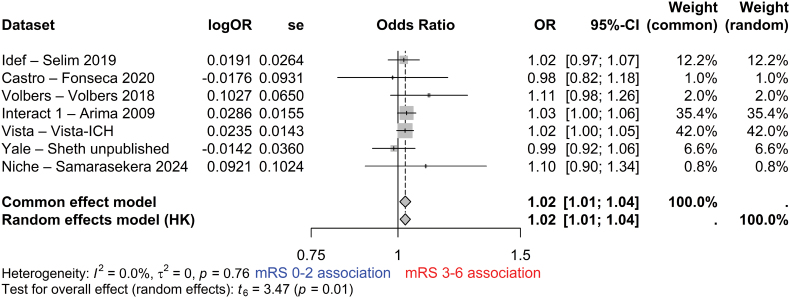
**Forest plot of the adjusted logistic regression model of the association between the change in absolute perihematomal edema volume between the diagnostic computed tomography (CT) and repeat CT at 72±12 hours after intracerebral hemorrhage onset and death or dependence at 90 days (n=495; after adjustment for age, sex, intracerebral hemorrhage volume, and intraventricular extension).** HK indicates Hartung-Knapp adjustment; mRS, modified Rankin Scale; OR, odds ratio; and VISTA, Virtual International Stroke Trials Archive.

In an unadjusted logistic regression analysis, growth in absolute PHE volume between the diagnostic CT and 72±12 hours after onset was associated with death or dependence (OR, 1.03 per mL [95% CI, 1.01–1.04]; I^2^=0%), with random and fixed-effects models producing the same result (Figure S2). This association remained after adjustment for age, sex, ICH volume, and intraventricular extension (OR, 1.02 per mL [95% CI, 1.01–1.04]; I^2^=0%) with random and fixed-effects models producing the same result (Figure 4).

In an unadjusted logistic regression model, absolute PHE volume at 24±12 hours after onset was associated with death or dependence in both random effects (OR, 1.05 per mL [95% CI, 1.03–1.07]; I^2^=20%) and fixed-effects (OR, 1.05 per mL [95% CI, 1.04–1.06]) models (Figure S3). This association did not remain in an adjusted random effects model (OR, 1.02 per mL [95% CI, 0.98–1.07]; I^2^=53%), but it persisted in a fixed-effects model (OR, 1.02 per mL [95% CI, 1.01–1.04]; Figure S4).

## Discussion

In this international individual participant data meta-analysis, absolute PHE growth assessed on CT in both the first 24 and 72 hours after ICH onset was associated with death or dependence at 90 days. We found that the increase in the odds of death or dependence at 90 days was 4% (95% CI, 1%–6%) for each l mL increase in PHE growth in the first 24 hours and 2% (95% CI, 1%–4%) in the first 72 hours after onset.

Two previous systematic reviews suggested that PHE growth was associated with worse outcomes.^6,7^ In the review that analyzed PHE growth by time^7^ worse functional outcome was associated with PHE growth in the first 72 hours but not with PHE growth in the first 24 hours after ICH onset; however, the 3 contributing studies were heterogeneous,^153,160,168^ and 90% of participants in these studies had deep ICH, which leads to less PHE growth than lobar ICH.^171^ Therefore, our findings provide additional support for quantifying PHE growth as early as 24 hours, or as late as 72 hours, for investigating associations with death or dependence at 90 days. Our findings also advance current research in this area by providing the first individual participant data meta-analytic justification for PHE as an imaging biomarker of outcome and at least a 24h time window for clinical trials of interventions targeting secondary injury to reduce PHE and reduce the risk of death or dependence after ICH.

Our study has some strengths. We included 66% of the available data and more than twice the number of participants with lobar ICHs in our primary outcome analysis than was possible in 2 recent systematic reviews,^6,7^ making our results applicable to both supratentorial deep and lobar ICH. The large sample size permitted multivariable analysis with adjustment for confounders. We adhered to a prespecified protocol and statistical analysis plan. We adjusted for ICH volume and intraventricular extension, which are closely related to Glasgow coma scale score (although we were unable to include Glasgow coma scale score in a multivariable analysis because this was not provided by 1 cohort^170^). Our results for the overall association between PHE growth and the primary outcome were consistent in unadjusted and adjusted analyses, in fixed-effects and random effects models, and consistent with a previous systematic review of aggregate data.^6^ The widespread availability of CT means that these findings are broadly applicable in a range of healthcare settings.

Our study has some limitations. The initial systematic review included studies published until November 17, 2021. We updated the literature search on August 19, 2025, and identified 5 new cohorts containing 586 participants,^174–178^ which might have been eligible for this individual participant data meta-analysis. However, the largest of these^174^ (n=246) is likely ineligible, because their data has been included in VISTA-ICH, so may have already been included in the individual participant data meta-analysis. Whether their data was definitely included in the data supplied by VISTA-ICH is uncertain because VISTA-ICH does not tell users which trials their data has come from.

We acknowledge the possibility of selection bias because 3 of the included cohorts were randomized trials, although this would not be expected to bias the association between PHE and outcome. Although only 18% of the shared individual participant data were used in the meta-analysis, characteristics of those included were similar to those who were not. We did not use aggregate data from cohorts which did not provide individual participant data; however, only 3 cohorts comprising 474 eligible participants provided a measure of the association between PHE growth in the first 24 hours and death or dependence which could have contributed to the primary outcome, and only 1 cohort provided a measure of the association between PHE growth in the first 72 hours and outcome (Supplemental Material). The IQR of ICH volumes in this data set was 6 to 21 mL, so we may not be able to make outcome predictions from the models for large hematoma volumes. We also did not adjust for ICH growth for 2 reasons. First, a previous meta-analysis of ICH growth showed a linear relationship between ICH volumes of this size on the diagnostic scan and the probability of growth,^179^ indicating that a change in ICH volume would likely be collinear with a change in PHE volume. Second, we wished to adhere to the prespecified statistical analysis plan. Although we sought to exclude participants treated with drugs that could affect PHE, most studies lacked these data. We were unable to explore the effect of newer imaging signs, such as PHE shape,^180^ or other variables, such as drugs with an inflammation-modulating effect, on the association between PHE and outcome, as we did not access participants’ scans, and few collaborating cohorts provided these data.

Whether blood pressure lowering modifies PHE and its association with outcome remains uncertain. Although we included participants in both the intervention and control arms of INTERACT 1 and 2, acute blood pressure lowering in INTERACT 1 did not affect PHE,^181^ making it unlikely that including the intervention arms from these trials affected the results. If it did, it may be more likely to reduce PHE, so this might have reduced the effect size of the association between PHE growth in the 0 to 24h period and death or dependency, rather than leading to a type 1 error. It was not feasible to do a sensitivity analysis by removing the intervention arms of the INTERACT 1 and 2 cohorts because both the intervention and control arms used the same range of antihypertensive medications, which were based on prespecified treatment protocols devised according to local availability of medications. The difference between the control and intervention arms was that the intensity of blood pressure lowering was greater in the intervention arm, but we did not have information on the intensity of blood pressure lowering received by individual participants in the 2 groups.

Although intensive blood pressure lowering in the second antihypertensive treatment of acute cerebral hemorrhage (ATACH-II) trial was associated with reduced PHE growth in the 24 hours after ICH onset,^160^ mean systolic blood pressures in the 2 hours after randomization were lower in the treatment arm of ATACH-II compared with INTERACT2 (128.9 vs 150 mm Hg, respectively), so extent of BP reduction may affect PHE and further investigation of the effects of intensive blood pressure lowering on PHE are warranted.

Our findings raise questions for future research into PHE. Future research should verify these findings in an independent data set and develop a prediction model to determine those at greatest risk of PHE growth after ICH. PHE growth beyond 72 hours after ICH onset may also be associated with death or dependence, but given the shortage of participants who had repeat imaging beyond 72 hours, this requires further exploration. We were unable to fully examine the PHE trajectory (including whether the trajectory of PHE differs by ICH location) because we lacked longitudinal data and only 2 published cohorts^34,171^ had these data. Future prospective studies should examine PHE evolution over time because it would further inform the duration of an intervention for PHE and help to target a PHE intervention at those most likely to benefit.

Our findings also have implications for the use of PHE as a potential imaging biomarker of outcome and treatment effect in randomized trials targeting secondary injury after ICH. Neuropathological studies show iron scavenging and apoptosis during the first 72 hours, with interleukin-1 and tumor necrosis factor production in perihematomal brain tissue; future studies could identify candidate drugs targeting these pathways.^182,183^

Previous studies of corticosteroids for ICH have not improved outcome after ICH,^184^ but studies have been limited by small sample sizes with heterogeneity in the dose, timing, and duration of therapy. Minimally invasive neurosurgery^185^ improves outcome after ICH, and this may in part be because of the removal of blood products, which can perpetuate secondary injury, therefore worsening PHE, but this requires further study.

Trials of interventions for PHE should consider repeat assessment of PHE within 72±12 hours, which may become feasible with the advent of both CT^186^ and magnetic resonance imaging^187^–based automated methods for measuring PHE. To date 2 small proof-of-concept trials have targeted PHE,^99,188^ of which one did not repeat brain imaging within 72 hours of ICH onset.^99^ Of 9 ongoing drug trials targeting PHE (accessed on March 15, 2025), 6 studies investigating Mirabegron (URL: https://www.clinicaltrials.gov; Unique identifier: NCT05369351), Celecoxib (URL: https://www.clinicaltrials.gov; Unique identifier: NCT05434065), Ir-CPI (*Ixodes ricinus* Contact Phase Inhibitor; URL: https://www.clinicaltrials.gov; Unique identifier: NCT05970224), MW01-6-189WH (a suppressor of inflammatory cytokine production; URL: https://www.clinicaltrials.gov; Unique identifier: NCT05020535), CN-105 (a neuroprotective peptide; URL: https://www.clinicaltrials.gov; Unique identifier: NCT06255977), and Edavarone (URL: https://www.clinicaltrials.gov; Unique identifier: NCT05953103) plan repeat imaging to assess PHE growth within 72 hours, and these could investigate proof of concept that PHE is an intermediate outcome of treatment effect.

These findings provide support for PHE as an imaging biomarker of functional outcome, and establish a time window of at least 24 hours for clinical trials of interventions targeting secondary injury to reduce PHE and the risk of death or dependence. In clinical practice, absolute PHE growth both in the first 24±12 and 72±12 hours might help to determine eligibility for randomized trials of treatments targeting secondary injury to reduce the global burden of death and disability attributable to ICH.

## ARTICLE INFORMATION

Presented in part at the European Stroke Organisation Conference, Munich, Germany, May 24–26, 2023, and Basel, Switzerland, May 15–17, 2024.

### Sources of Funding

### Disclosures

Dr Samarasekera reports grants from the National Health Service (NHS) Research Scotland and grants from Stroke Association. Dr Volbers reports personal fees from Pfizer AG/Bristol Myers Squibb SA, personal fees from Bayer AG, personal fees from Ipsen Pharma, personal fees from CSL Behring, personal fees from Pfizer AG/Bristol Myers Squibb SA, personal fees from AstraZeneca, nonfinancial support from AbbVie, outside the submitted work. Dr Selim reports grants from National Institutes of Health (NIH)/National Institute of Neurological Disorders and Stroke (NINDS) and National Institute on Aging (NIA; U01NS102289; UF1NS120871; UG3NS128397); royalties for UpToDate Inc, authored a section on secondary prevention of ICH; and Oxford University Press; compensation from Bioxodes Inc for consultant services; compensation from EMVision Medical Devices Ltd for consultant services; grants from NIH/NIA; stock holdings in NeuGel Inc; compensation from Alnylam Pharmaceuticals for data and safety monitoring services; compensation from MedRhythms, Inc for consultant services; and compensation from AegisCN, LLC for consultant services. Dr Sheth reports a patent pending for Stroke wearables licensed to Alva Health; compensation from Philips for data and safety monitoring services; grants from Genentech Inc; compensation from Bexorg for consultant services; stock options in BrainQ; compensation from Rhaeos for consultant services; grants from Hyperfine; and compensation from Astrocyte for consultant services. Dr Mair reports grants from British Heart Foundation; compensation from Canon Medical Systems Corporation for consultant services; and grants from Medical Research Council. Dr Moullaali reports grants from Scottish Heart and Arterial Risk Prevention (SHARP). Dr Parry-Jones reports compensation from Alveron for consultant services. Dr Sprigg reports grants from National Institute for Health Research. Dr Schreuder reports grants from the Netherlands Heart Foundation; grants from Swedish Orphan Biovitrum AB; and grants from ZonMw. Dr Steiner reports compensation from Daiichi Sankyo Company for consultant services; compensation from AstraZeneca for consultant services; compensation from Bristol Myers Squibb for consultant services; compensation from Bayer for consultant services; compensation from Chiesi USA Inc for other services; and compensation from Boehringer Ingelheim for consultant services. Dr Butcher reports compensation from AstraZeneca Australia for consultant services and compensation from Boehringer Ingelheim for consultant services. Dr Hanley reports stock options in EpiWatch; compensation from HiCatalyst for consultant services; and gifts from Jeffrey and Harriet Legum Professorship in Acute Neurological Medicine at Johns Hopkins University. Dr Ziai reports grant support from the National Institute of Neurological Disorders and Stroke (NINDS) and compensation from Neurocritical Care Society for consultant services. Dr Demchuk reports grants from SFJ Pharmaceuticals to other; compensation from Boehringer Ingelheim for consultant services; compensation from NovaSignal for consultant services; compensation from Medtronic for consultant services; compensation from Lumosa for data and safety monitoring services; a patent issued for Stroke imaging software licensed to Circle NVI; stock holdings in Circle NVI; compensation from Hoffmann-La Roche Limited for consultant services; compensation from Philips for data and safety monitoring services; grants from AstraZeneca; compensation from AstraZeneca Canada for other services; grants from Sense Neuro Diagnostics to other; and compensation from Novo Nordisk AS for other services. Dr Weir reports grants from the British Heart Foundation.

Dr R.A.-S. Salman reports grants paid to his institution from The Stroke Association, British Heart Foundation and Chief Scientist Office of the Scottish Government Health Department; consulting fees paid to his institution from Recursion Pharmaceuticals; honoraria paid to their institution from European Stroke Masters (European Stroke Organisation); participation on Novo Nordisk NN9931-4553 and NN9931-4554 end point adjudication committee; and personal payment as clinical director of UK Clinical Research Collaboration network of registered Clinical Trials Units. The other authors report no conflicts.

### Supplemental Material

Tables S1–S9

Figures S1–S4

Protocol

Statistical Analysis Plan

## Supplementary Material


