# Supplementary material for: Perihematomal Edema and Functional Outcome After Intracerebral Hemorrhage: A Meta-Analysis of Individual Participant Data
Source: Stroke. 2026 Mar 4;57(5):1310–24. doi: 10.1161/STROKEAHA.125.053991 (PMC13117555; doi:10.1161/STROKEAHA.125.053991)
Supplement: Supplementary file 1 [file str-57-1310-s001.pdf]

SUPPLEMENTAL MATERIAL. Association between perihaematomal oedema and functional outcome after stroke due to intracerebral haemorrhage: a systematic review and meta-analysis of individual participant data: supplement

Title	Page
Table S1 Study characteristics of eligible cohorts which did not provide individual participant data	2
Table S2 Study characteristics of included cohorts	3
Table S3 IPD provided by each cohort for the analysis of the association between the change in PHO volume 24 hours after ICH onset and outcome	4
Table S4 IPD provided by each cohort for the analysis of the association between the change in PHO volume 72 hours after ICH onset and outcome	5
Table S5 Characteristics of participants included in the assessment of change in absolute PHO volume during 0-72 h by functional outcome assessed by modified Rankin scale (mRS) at 90 ± 14 days; mRS scores 3-6 indicate poor outcome	6
Table S6 Risk of bias in cohorts which supplied IPD and were included in the meta-analysis	7
Table S7 Risk of bias in cohorts which supplied IPD but were not included in the meta-analysis	8
Table S8 Characteristics of participants who were included in the individual participant data meta-analysis (n=1523) vs. those whose data were supplied but were excluded (n=6846)	9
Table S9 ICH volumes, absolute PHO volumes and ICH: absolute PHO volume ratio on scans done 24 ± 12 hours after ICH symptom onset	10
Figure S1: Forest plot of the unadjusted logistic regression model of the association between the change in absolute PHO volume between diagnostic CT and repeat CT at 24 ± 12 hours after intracerebral haemorrhage onset and death or dependence at 90 days	11
Figure S2: Forest plot of the unadjusted logistic regression model of the association between the change in absolute PHO volume between the diagnostic CT and repeat CT at 72 ± 12 hours after intracerebral haemorrhage onset and death or dependence at 90 days	12
Figure S3: Forest plot of the unadjusted regression model of the association between absolute PHO volume at 24 ± 12 hours after ICH symptom onset and death or dependence at 90 days	13
Figure S4: Forest plot of the adjusted regression model of the association between absolute PHO volume at 24 ± 12 hours after ICH symptom onset and death or dependence at 90 days	14
Protocol	15
Statistical analysis plan	25

Table S1: Study characteristics of eligible cohorts which did not provide individual participant data

Study (year)	Sample size	Outcome measure	Timing of assessment of outcome after ICH onset	Odds ratio for PHO growth in first 24 hours after ICH onset and death or dependence[1] at 90 days	Odds ratio for PHO growth in first 72 hours after ICH onset and death or dependence[1] at 90 days
Inaji (2003)	14	NIHSS[2]	in hospital	×	×
Sansing (2003)	80	mRS[3]	not stated	×	×
Leira (2004)	266	Canadian stroke scale	in hospital	×	×
Alvarez Sabin (2004)	21	mortality	90 days	×	×
Delgado (2006)	78	mRS	90 days	×	×
Sansing (2011)	287	mRS	90 days	×	×
Feng (2012)	135	mRS	90 days	×	×
Li (2013)	59	mRS	90 days	×	×
Bakhshayesh (2014)	63	mRS	90 days	×	×
Suarez-Pinilla (2014)	56	mortality, mRS	in hospital, discharge, three months after discharge	×	×
Urday (2016)	110	mRS	90 days	✓	✓
Roy O'Reilly (2017)	51	mRS	90 days	×	×
Rendevski (2018)	50	Canadian stroke scale	90 days	×	×
Chen (2019)	131	mRS	90 days	✓	×
Leasure (2019)	780	mRS	90 days	×[4]	×
Peng (2019)	121	mRS	discharge	×	×
Zang (2019)	59	NIHSS, Barthel index	90 days	×	×
Halstead (2020)	166	mRS, mortality	discharge	×	×
Li (2020)	98	mRS,	two weeks	×	×
Huan (2021)	159	mRS	90 days	×	×
Kashiwazaki (2021)	143	NIHSS	two weeks	×	×
Kim (2021)	66	mRS	discharge	×	×
Lv (2021)	233	mRS	90 days	✓	×
Tan (2021)	60	mRS, mortality	one year	×	×
Zhang (2021)	635	mRS	90 days	×	×
Zhao (2021)	20	mRS	90 days	×	×

[1]Death or dependence = modified Rankin scale 3-6
[2]NIHSS - National Institute for Health Stroke Scale
[3]mRS – modified Rankin scale
[4]ATACH-2 trial: Odds ratio for association between PHO growth in the 24 hours after ICH onset and outcome in control arm only not provided (publication provides an odds ratio for the association after combining both intervention and control arms).

Table S2: Study characteristics of included cohorts

Author (year)	Sample size	Prospective recruitment	Pre-specified scanning protocol	ICH onset criteria	Time point of measuring PHO	Method of measuring PHO and ICH vol	PHO measure	Units used for PHO measurement	Time from sx onset to baseline scan, hours	Timepoint of measuring functional outcome	Assessment of functional outcome	Surgery	BP lowering intervention	Osmotic agent used,n
Study characteristics of **published** cohorts														
Yang (2015)- INTERACT 1 and 2 trials	1138	Y	Y	<6h[1]	Admission, 24h	planimetric	absolute change in PHO volume	mL	1.8 (1.2-2.6)	90 days	mRS	excluded	Guideline group BP BP>180, Intensive group BP<140	382 received mannitol
Fonseca (2019)	135	N	N	<24h[2]	Admission, 24-36h after onset	manual	absolute	NS	3.2 (1.8-14.2)	90 days	mRS	Included EVD but excluded other surgery	NS	NS
Sprugel (2019)	300	Y	N	NS	Admission, days 1, 2–3, 4–6, 7–9, 10–12, 13–15, 16–18, and 19–22	planimetric	absolute	mL	NS	90 days	mRS	NS	NS	0 (excluded)
Selim (2019)	147 (placebo arm)	Y	Y	<24h[1]	Admission, post infusion (at least 72h after onset)	planimetric	relative	mL	NS	90 days	mRS	Excluded surgical evacuation before admin of intervention	NS	NS
Shirazian (2021)	446	Y	N	<12h[1]	admission, 12–24h from admission, 36–48 h after ICH	ABC/2	Absolute change in volume	mL	NS	30days 90days	30 day mortality, 90 day mRS	Excluded if underwent surgery before second scan	NS	NS
Samarasekera 2024	106	Y	Y	<72h	Admission, days 3, 7 & 14 after onset	planimetric	absolute	mL	2.8 (1.4-8.4)	90d	mRS	None	to target 130-140 systolic	none
Study characteristics of Yale cohort **(unpublished)**														
Sheth et al.	221	N	N	<24h	Admission, repeat scans at median 49h after onset (IQR 34-67)	planimetric	absolute	mL	5.2 (3.4-8.6)	90d	mRS	22 (10%) had surgery- these participants were excluded	NS	NS

Y = yes, N = no, NS=not stated, all studies hospital based, h= hours, mL= millilitres, mRS = modified Rankin scale, [1]: does not state how cases where onset time cannot be established, were considered, [2]: dataset classifies time of onset as awake at onset, last seen well and awoke from sleep

Table S3: IPD provided by each cohort for the analysis of the association between the change in PHO volume 24 hours after ICH onset and outcome (N/A =not applicable)

Study	Participants provided	Not spontaneous ICH	First CT head done >72 hours after ICH onset	Had surgery	Repeat CT head done >14 days after ICH onset	Did not have a repeat CT head 24±12 hours after ICH onset	Not included in published study	PHO volume missing on diagnostic scan	PHO volume missing on interval scan	PHO measurement not suitable	Modified Rankin score missing	Modified Rankin score assessed at a different time point (not at 90±14 days)	Data did not fulfil consistency checks	Sample size contributing to IPD was too small	Participants included in meta-analysis
Castro (2019)	135	0	7	51	1	61	0	0	0	0	0	0	0	15	0
Gioia (2015)	75	0	0	2	0	24	2	0	0	0	19	0	0	28	0
Gusdon (2020)	216	0	0	0	0	0	0	0	0	0	0	0	216	0	0
Hanley (2019)	499	0	0	250	2	0	0	0	0	0	0	247	0	0	0
Iglesias-Rey (2018)	887	0	0	0	0	0	0	887	0	0	0	0	0	0	0
Rodriguez-Luna (2016)	390	0	0	0	0	0	0	0	0	390	0	0	0	0	0
Samarasekera (2024)	106	0	0	0	5	93	0	0	0	0	0	0	0	8	0
Selim (2019)	148	0	0	38	0	108	0	0	0	0	2	0	0	0	0
Shirazian (2021)	233	0	0	0	0	0	0	0	0	0	0	0	0	0	233
Sprugel (2019)	275	0	7	93	0	91	0	0	0	0	0	0	0	0	84
VISTA ICH	938	27	11	196	248	0	N/A	191	128	0	24	0	0	0	113
Wu (2017)	1013	0	0	0	0	0	0	0	0	0	0	1013	0	0	0
Yale cohort	221	0	0	22	4	110	N/A	0	0	0	38	0	0	0	47
Yang (2015) -Interact 1 cohort	404	0	0	0	3	48	58	25	0	0	11	42	0	0	217
Yang (2015) -Interact 2 cohort	2829	0	0	0	1	10	1865	101	8	0	12	178	1	0	653

Table S4: IPD provided by each cohort for the analysis of the association between the change in PHO volume 72 hours after ICH onset and outcome (N/A =not applicable)

Study	Participants provided	Not spontaneous ICH	First CT head done >72 hours after ICH onset	Had surgery	Repeat CT head done >14 days after ICH onset	Did not have a repeat CT head 72±12 hours after ICH onset	Not included in published study	PHO volume missing on diagnostic scan	PHO volume missing on interval scan	PHO measurement not suitable	Modified Rankin score missing	Modified Rankin score assessed at a different time point (not at 90±14 days)	Data did not fulfil consistency checks	Participants included in meta-analysis
Castro (2019)	135	0	7	51	1	58	0	0	0	0	0	0	0	18
Gioia (2015)	75	0	0	2	0	52	2	0	0	0	19	0	0	0
Gusdon (2020)	216	0	0	0	0	0	0	0	0	0	0	0	216	0
Hanley (2019)	499	0	0	250	2	0	0	0	0	0	0	247	0	0
Iglesias-Rey (2018)	887	0	0	0	0	0	0	887	0	0	0	0	0	0
Rodriguez-Luna (2016)	390	0	0	0	0	0	0	0	0	390	0	0	0	0
Samarasekera (2024)	106	0	0	0	5	79	0	0	0	0	0	0	0	22
Selim (2019)	148	0	0	38	0	68	0	0	0	0	2	0	0	40
Shirazian (2021)	233	0	0	0	0	233	0	0	0	0	0	0	0	0
Sprugel (2019)	275	0	7	93	0	140	0	0	0	0	0	0	0	35
VISTA ICH	938	27	11	196	248	0	N/A	191	126	0	24	0	0	115
Wu (2017)	1013	0	0	0	0	0	0	0	0	0	0	1013	0	0
Yale cohort	221	0	0	22	4	128	N/A	0	0	0	38	0	0	29
Yang (2015) -Interact 1 cohort	404	0	0	0	3	29	58	25	0	0	11	42	0	236
Yang (2015) -Interact 2 cohort	2829	0	0	0	1	671	1865	101	0	0	12	178	1	0

Table S5: **Characteristics of participants included in the secondary analysis of PHO growth between 0-72h by functional outcome assessed by modified Rankin scale (mRS) at 90 ± 14 days; mRS scores 3-6 indicate poor outcome**

	**Overall n= 495**	**Good outcome (mRS 0-2) n= 233**	**Poor outcome (mRS 3-6) n= 262**
Sex, female, n (%)	195 (39%)	78 (33%)	117 (45%)
Age, years, median (IQR)	66 (55-74)	62 (52-70)	69 (59-78)
Glasgow coma scale on admission, median (IQR)[1]	15 (13-15)	15 (14-15)	14 (12-15)
Intracerebral haemorrhage location			
supratentorial lobar	78 (16%)	35 (15%)	43 (16%)
supratentorial deep	287 (58%)	147 (63%)	140 (54%)
infratentorial	31 (6.3%)	17 (7·3%)	14 (5·4%)
unknown	99 (20%)	34 (15%)	65 (25%)
Intracerebral haemorrhage volume on the diagnostic CT, mL, median (IQR)[2]	9 (4-20)	7 (3-12)	13 (5-28)
PHO volume on the diagnostic scan, mL, median (IQR)	8 (4-16)	7 (3-12)	10 (4-21)
Intraventricular extension, n (%)	152 (31%)	45 (19%)	107 (41%)
Time interval from symptom onset to diagnostic CT (h), median (IQR)	2·4 (1·4-5.4)	2·3 (1·5-4·2)	2·4 (1·4-7.9)
Time from symptom onset to second CT (h), median (IQR)	73 (72-76)	73 (72-76)	73 (72-76)
Intracerebral haemorrhage volume on second CT, mL, median (IQR)[1]	10 (5-24)	7 (4-14)	16 (7-34)
PHO volume on CT at 72± 12 hours, mL, median (IQR)[1]	13 (6-26)	11 (5-19)	18 (8-34)

[1]GCS missing in two participants: one in the good outcome and one in the poor outcome group.
[2]ICH volume missing in one participant in the poor outcome group.

Table S6: Risk of bias in cohorts which supplied IPD and were included in the meta-analysis

(if the study did not report an association between PHO and functional outcome, item listed as not applicable)
Justification– Items with a low risk of bias in green; moderate risk of bias in orange; each study given an overall risk of bias shown by their colour in the column, titled 'Study'. Most important domains marked with *

	Study participation*	Justification	Study attrition	Justification	Prognostic factor measurement*	Justification	Functional outcome measurement*	Justification	Study confounding	Justification	Statistical analysis and reporting	Justification
Fonseca (2020) [orange]	[orange]	Selection bias (retrospective hospital-based cohort)	[orange]	Not reported	[green]	Blinded assessment of PHO in relation to clinical information, valid and reliable measurement	[orange]	Same outcome for all participants, but method of ascertainment not reported	[green]	Adequate adjustment of confounders	[green]	Appropriate analysis
Samarasekera (2024) [orange]	[orange]	Selection bias (milder ICH)	[orange]	13% did not complete study	[green]	Blinded assessment of PHO, valid and reliable measurement	[green]	Same for all participants	[orange]	Incomplete adjustment for confounders	[green]	Appropriate analysis
Selim (2019) [green]	[orange]	Selection bias likely since it is a trial cohort	[green]	Participants lost to follow up described, unlikely to bias findings	[green]	Blinded assessment of PHO, valid and reliable measurement	[green]	Same for all participants		Not applicable	[green]	Appropriate analysis
Shirazian (2021) [orange]	[orange]	Retrospective analysis of prospective single centre registry	[green]	No loss to follow up	[green]	Blinded assessment of PHO, valid and reliable measurement	[orange]	Outcome assessed by review of medical records	[orange]	Incomplete adjustment for confounders	[green]	Appropriate analysis
Sprugel (2019) [orange]	[orange]	Retrospective analysis of prospective single centre registry	[orange]	Not described	[green]	Blinded assessment of PHO, valid and reliable measurement	[green]	Same for all participants	[green]	Adequate adjustment for confounders	[green]	Appropriate analysis
Yang (2015) [orange]	[orange]	Retrospective analysis of prospective trial cohort	[green]	No loss to follow up reported	[green]	Blinded assessment of PHO, valid and reliable measurement	[green]	Same for all participants	[green]	Adequate adjustment for confounders	[green]	Appropriate analysis

Table S7: Risk of bias in cohorts which supplied IPD but were not included in the meta-analysis

(if the study did not report an association between PHO and functional outcome, item listed as not applicable)
Justification– Items with a low risk of bias in green; moderate risk of bias in orange; each study given an overall risk of bias shown by their colour in the column, titled 'Study'. Most important domains marked with *

Study	**Study participation***	**Justification**	**Study attrition**	**Justification**	**Prognostic factor measurement***	**Justification**	**Functional outcome measurement***	**Justification**	**Study confounding**	**Justification**	**Statistical analysis and reporting**	**Justification**
Gioia (2015)		Selection bias likely since it is a trial cohort		No loss to follow up		Blinded assessment of PHO in relation to clinical information, valid and reliable measurement		Same for all participants		Not applicable		Appropriate analysis
Gusdon (2020)		Selection bias (retrospective hospital-based cohort)		Not reported		Unclear when CT head scans were done in relation to ICH onset or if PHO was assessed blind to case vs. control status or outcome		Adjudication of clinical outcome was not the same for case vs. control groups		Incomplete adjustment of confounders		Appropriate analysis
Hanley (2019)		Selection bias likely since it is a trial cohort		Participants lost to follow up described, unlikely to bias findings		Blinded assessment of PHO in relation to clinical information, valid and reliable measurement		Same for all participants		Not applicable		Appropriate analysis
Iglesias-Rey (2018)		Selection bias (retrospective analysis of prospective registry)		Not reported		Blinded assessment of PHO in relation to clinical information, valid and reliable measurement		Same clinical outcome for all participants, but method of ascertainment not reported		Incomplete adjustment of confounders		Appropriate analysis
Rodriguez-Luna (2016)		Multicentre prospective cohort study (PREDICT study)		Participants lost to follow up described, unlikely to bias findings		Blinded assessment of PHO in relation to clinical information, valid and reliable measurement		Not applicable – primary outcome was haematoma expansion		Not applicable		Appropriate analysis
Wu (2017)		Selection bias (retrospective cohort)		1% lost to follow up, unlikely to bias findings		Assessment of oedema described but oedema trajectory modelled by assuming same proportional growth to 72 hours		Same for all participants		Adequate adjustment of confounders		Appropriate analysis

Table S8: Characteristics of participants who were included in the individual participant data meta-analysis (n=1523) vs. those whose data were supplied but were excluded (n=6846)

	Participants included (n=1523)	Participants excluded (n=6846)
Sex, female, n (%)[1]	589 (39%)	2,738 (40%)
Age, years, median (IQR)[2]	66 (55-75)	66 (56-76)
Glasgow coma scale on admission, median (IQR)[3]	14 (13-15)	14 (11-15)
Intracerebral haemorrhage location		
supratentorial lobar	308 (20)	1801 (26)
supratentorial deep	1022 (67)	3883 (57)
infratentorial	91 (6)	481 (7)
unknown	102 (7)	632 (9)
Exclusively intraventricular haemorrhage	0 (0)	49 (1)
Intracerebral haemorrhage volume on the diagnostic CT, mL, median (IQR)[4]	11 (6-21)	15 (7-33)
Intraventricular extension, n (%)[5]	484 (32)	2259 (33)

[1]Data missing in three participants who were excluded
[2]Data missing in 4 participants who were excluded
[3]Data missing in 240 participants who were included and 1035 participants who were excluded
[4]Data missing in 1 participant who was included and 525 participants who were excluded
[5]Data missing in 20 participants who were excluded

Table S9: ICH volumes, absolute PHO volumes and ICH volume: absolute PHO volume ratio on scans done 24 ± 12 hours after ICH symptom onset; values are median (IQR); the ratio is calculated from the actual ICH volumes and PHO volumes for each participant rather than the median ICH volumes and PHO volumes for each cohort.

**Cohort**	**Entire dataset**	**Sprugel (2019)**	**INTERACT 1 (Arima 2009)**	**INTERACT 2 (Anderson, 2013)**	**Shirazian (2021)**	**VISTA -ICH**	**Sheth et al. (unpublished)**
Number of patients	1,344	84	217	651	232	113	47
ICH volume	12.8 (6.6-25.0)	18.9 (6.0-35.2)	9.9 (4.8-19.2)	11.4 (5.9-20.4)	19.2 (10.3-33.2)	13.5 (4.8-29.2)	16.4 (7.2-37.4)
PHO volume	6.8 (2.8-16.1)	18.4 (10.6-30.7)	11.2 (5.3-19.7)	3.4 (1.6-6.0)	15.5 (9.2-25.2)	11.6 (5.6-31.0)	10.7 (5.2-28.2)
ICH: PHO volume ratio	1.8 (0.9-3.5)	0.8 (0.5-1.3)	0.9 (0.6-1.6)	3.3 (2.1-5.6)	1.2 (0.8-1.8)	1.0 (0.7-1.3)	1.2 (0.8-2.0)
Missing	3	0	0	2	1	0	0

Figure S1: Forest plot of the unadjusted logistic regression model of the association between the change in absolute PHO volume between diagnostic CT and repeat CT at 24 ± 12 hours after intracerebral haemorrhage onset and death or dependence at 90 days (n=1347).
Weight (common) describes how much a given study contributes to the overall effect under a common-effect (fixed effect) model.
Weight (random) describes how much a given study contributes to the overall effect under a random effects model accounting for between study heterogeneity.

OR= odds ratio, CI = confidence interval, HK = Hartung-Knapp adjustment, mRS = modified Rankin Scale, se = standard error

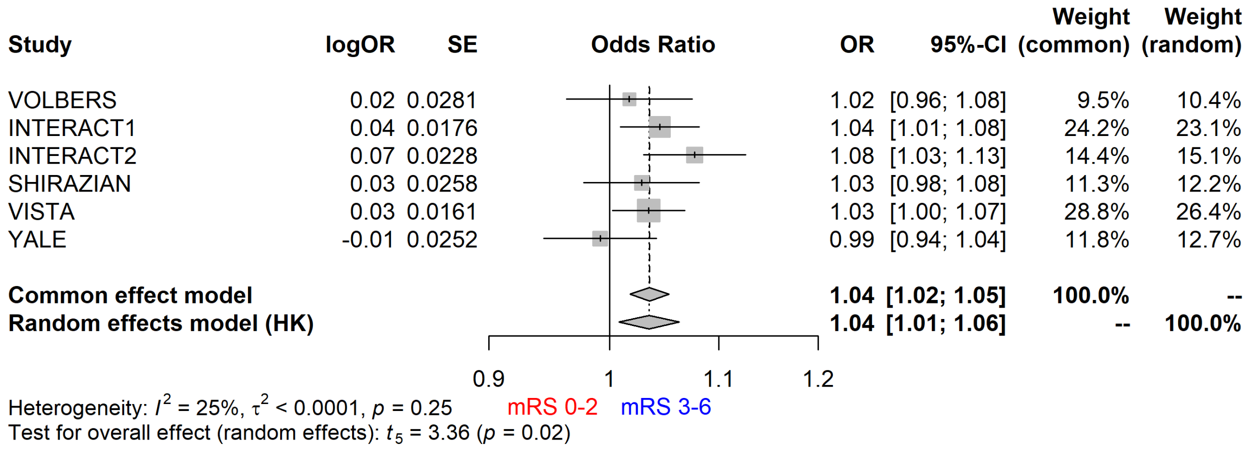

Figure S2: Forest plot of the unadjusted logistic regression model of the association between the change in absolute PHO volume between the diagnostic CT and repeat CT at 72 ± 12 hours after intracerebral haemorrhage onset and death or dependence at 90 days (n=495)

Weight (common) describes how much a given study contributes to the overall effect under a common-effect (fixed effect) model.
Weight (random) describes how much a given study contributes to the overall effect under a random effects model accounting for between study heterogeneity.

OR= odds ratio, CI = confidence interval, HK = Hartung-Knapp adjustment, mRS = modified Rankin Scale, se = standard error

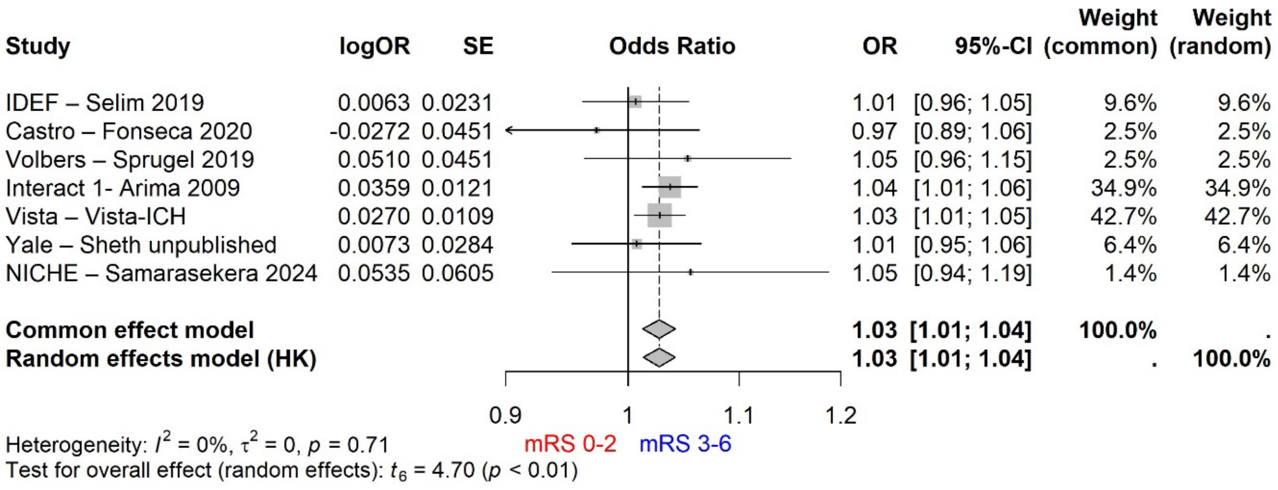

Figure S3: Forest plot of the unadjusted regression model of the association between absolute PHO volume at 24 ± 12 hours after ICH symptom onset and death or dependence at 90 days

Weight (common) describes how much a given study contributes to the overall effect under a common-effect (fixed effect) model.

Weight (random) describes how much a given study contributes to the overall effect under a random effects model accounting for between study heterogeneity.

OR= odds ratio, CI = confidence interval, HK = Hartung-Knapp adjustment, se = standard error

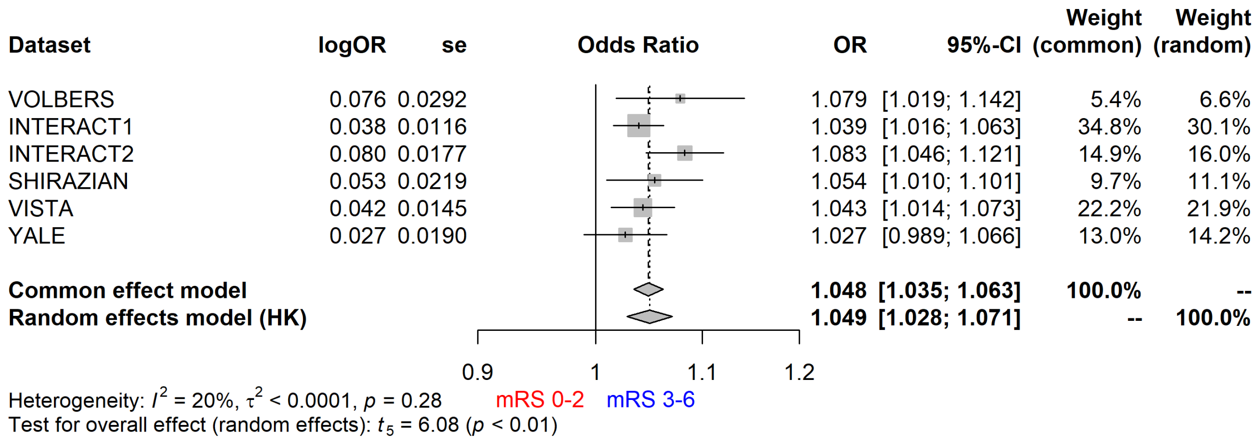

Figure S4: Forest plot of the adjusted regression model of the association between absolute PHO volume at 24 ± 12 hours after ICH symptom onset and death or dependence at 90 days (after adjustment for age, sex, ICH volume and intraventricular extension)

Weight (common) describes how much a given study contributes to the overall effect under a common-effect (fixed effect) model.

Weight (random) describes how much a given study contributes to the overall effect under a random effects model accounting for between study heterogeneity.

OR= odds ratio, CI = confidence interval, HK = Hartung-Knapp adjustment, se = standard error

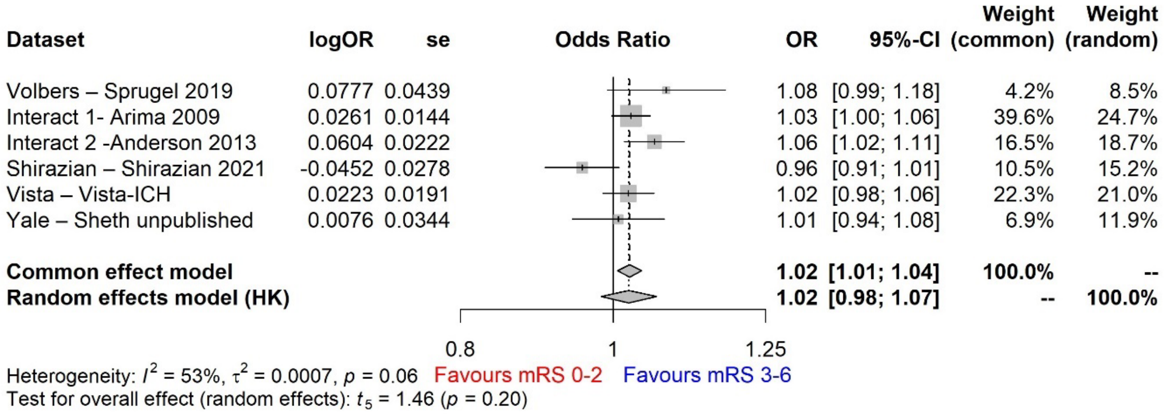

The association between peri-haematomal oedema and functional outcome after intracerebral haemorrhage - Individual participant data meta-analysis (this protocol has been published on PROSPERO https://www.crd.york.ac.uk/PROSPERO/view/CRD42021253263)

Authors

Dr N Samarasekera[1]

Dr G Mair[1]

Professor C Weir[2]

Dr X Wang[3]

Dr T Moullaali[1]

Dr A Parry-Jones[4]

Mr J Drever[1]

Mrs Sharon Tuck[2]

Professor R Al-Shahi Salman[1,2]

Institutional affiliations

1. Centre for Clinical Brain Sciences, University of Edinburgh, 49 Little France Crescent, Edinburgh, UK, EH16 4SB
2. Edinburgh Clinical Trials Unit, Usher Institute, University of Edinburgh, 49 Little France Crescent, Edinburgh, UK, EH16 4SB
3. The George Institute for Global Health, Faculty of Medicine, University of New South Wales, Sydney, New South Wales, Australia
4. Institute of Cardiovascular Sciences, University of Manchester, Manchester, UK

Corresponding author

Dr N Samarasekera (email: neshika.samarasekera@ed.ac.uk)

Author contributions

NS and RASS conceived the study. All authors were responsible for obtaining funding for the review. NS was responsible for first draft of the protocol, and all authors provided critical review of the current protocol. XW, ST and CJW are responsible for statistical oversight.

Guarantor of review

Dr N Samarasekera

Funding

This review is funded by a British Heart Foundation Research Excellence award to the University of Edinburgh (RE/18/5/34216). NS is supported by an NHS Scotland Research fellowship. GM is supported by the Stroke Association Edith Murphy Foundation Senior Clinical Lectureship. Potential studies for inclusion have reported

their own funding sources. The review sponsor is the University of Edinburgh, UK. The funder and sponsor had no role in the development of the protocol.

Background

Intracerebral haemorrhage (ICH) accounts for approximately 10% of strokes in high income countries and 20% of strokes in low/middle income countries.[1] Approximately 40% of patients die within the first month, and 86% are dead or dependent within one year.[2] Haemostatic drugs, blood pressure lowering and haematoma evacuation for supratentorial ICH have not clearly improved outcome, so there is still no effective acute treatment for ICH.

Peri-haematomal oedema (PHO) is visible around ICH on computed tomography (CT) and magnetic resonance imaging (MRI) in most patients. On CT, PHO appears as a hypoattenuated area around the ICH and on T2-weighted MRI it appears as hyperintense area. PHO results from red blood cell breakdown, thrombin accumulation and neuro-inflammation. It contributes to mass effect and is a promising potential therapeutic target after ICH.[3] 'Treating the global and secondary effects of ICH including oedema formation' is a clinical research priority set by The Second Haemorrhagic Stroke Academia Industry (HEADS-2) consortium in 2020,[4] and by The Stroke Association's Haemorrhagic Stroke Review.[5] However, the HEADS-2 consortium also acknowledged 'the debatable relationship between peri-haematomal oedema and functional outcome.' Knowledge of factors which might modify this association would help to stratify patients according to their risk of PHO, to help select participants for trials of treatments targeted at PHO.

In a systematic review of 6681 articles on 4 April 2021 we identified 44 studies (using CT [n=35], MRI [n=7], both CT and MRI [n=2]) of 8,374 participants, which assessed both PHO and functional outcome in adults after spontaneous ICH. Of 37 studies which examined the association between PHO and functional outcome, 30 studies found modest associations between PHO and worse outcomes, three found that PHO was associated with better outcomes and four found no association. Only eight studies adjusted for other variables known to affect outcome after ICH,[6-13] and a meta-analysis of five[9-13] of these studies that assessed outcome using the modified Rankin scale at 90 days did not find an association between PHO and outcome (odds ratio [OR] 1.05, 0.99-1.11). However, the existence, strength, direction, and modifiers of an association remain uncertain, partly because of heterogeneity between these five studies, including the time of PHO assessment in relation to ICH onset (admission [n=1][10] over first 24 hours [n=2],[9,11] over first 72 hours [n=1][13] and within first 12 days [n=1][12]).

Although PHO within 24 hours of ICH onset may be associated with poorer outcome,[14] the strength of the association and its existence when PHO is measured beyond 24 hours are unclear. Little is known about other variables which might modify the association between PHO and outcome. Sex,[15] blood pressure, hyperglycaemia and statin use have all been suggested as potential effect modifiers,[16] but variables have rarely been studied more than once, findings have been inconsistent, and small sample sizes and heterogeneity both in PHO measurement and timing of brain imaging following ICH have precluded firm conclusions.

Because the differences between studies preclude standard meta-analysis based on aggregated data we intend to use an individual participant data meta-analysis (IPDMA) to (1) explore the strength of the association between PHO and outcome after ICH and (2) to identify variables which may modify the strength of the association of PHO with outcome.

Hypothesis

Our hypothesis is that PHO is likely to affect functional outcome after ICH and that the time point of measurement and clinical variables such as age and blood pressure on admission may modify this association.

Research questions

1. In adults who have a spontaneous ICH:
    a. Is the change in PHO volume between two selected time points in the first two weeks after ICH associated with longer term functional outcome (for example, three months after ICH onset)?
    b. Is the volume of PHO at a certain time point in the first two weeks after ICH associated with longer term functional outcome (for example, three months after ICH onset)?
2. How does the association between PHO and functional outcome vary
    a. according to the time points between which change in PHO volume is measured in the first two weeks after ICH onset?
    b. according to the time at which PHO volume is measured in the first two weeks after ICH onset?
3. Is the association between PHO volume and functional outcome, as outlined in 1a affected by:
    a. clinical variables such as participant age, blood pressure on admission?
    b. radiological variables such as ICH volume or ICH location?
4. Is the association between change in PHO volume and functional outcome, as outlined in question 1b affected by:
    a. clinical variables such as participant age, blood pressure on admission?
    b. radiological variables such as ICH volume or ICH location?

Methods

Research design

We will conduct an individual participant data meta-analysis.

Study identification

On 4 April 2021 we completed a comprehensive search of electronic databases (Embase (1980-) and Medline (1950-); appendix 1), hand searched bibliographies of relevant studies, and performed forward citation searching for each included article by using Google Scholar to identify relevant studies (registered on PROSPERO CRD42020157088). We excluded conference abstracts.

Inclusion and exclusion criteria

Study level inclusion criteria:

- Acute spontaneous (non-traumatic) supratentorial or infratentorial ICH confirmed by CT brain imaging.
- Repeat CT brain imaging performed at least once up to 14 days after the first brain imaging study.
- Observational cohort studies or control arms of randomised trials that measured PHO and functional outcome (using modified Rankin scale or similar measure) after ICH.
- Randomised trials which measured PHO and functional outcome (using modified Rankin scale or similar measure) after ICH, where the intervention used should not affect PHO; or the control arm of randomised trials only if the intervention may have affected PHO (for example, steroids, mannitol, hypertonic saline).

Exclusion criteria:

Study level:

- Studies reporting participants who are included in other publications that reported a larger cohort.
- Studies solely using MRI to assess PHO (since CT is the most frequently used imaging modality for participants with ICH and is reliable for the semiautomated assessment of PHO)[17]
- Studies involving <5 participants with spontaneous ICH

Participant level:

- Age less than 18 years.
- Secondary causes of ICH (including trauma, tumour, intracranial aneurysm, arteriovenous malformation, arteriovenous fistula, cavernous malformation, venous thrombosis, moyamoya syndrome, reversible cerebral vasoconstriction syndrome, or haemorrhagic transformation of ischaemic stroke).
- Exclusively intraventricular/subarachnoid/subdural/extradural haemorrhage.
- Participants treated with surgery, mannitol or steroids following their ICH (since these might also affect PHO)
- Participants where first (diagnostic) CT was performed more than 72 hours after ICH onset or where the time from ICH onset to first CT was unknown.
- Participants who might be eligible for inclusion in the IPDMA but whose data were not originally published in the specific study.

Data collected

Study level variables

- First author of study and date
- Study period (years)
- Country
- Funding source
- Participants identified prospectively vs. retrospectively

- Inclusion and exclusion criteria (for example age range, admission blood pressure, GCS)
- Prespecified CT scanning protocol used
- Prospective outcome ascertainment used
- Time point of measuring functional outcome and method such as modified Rankin Scale
- Number of participants provided by each study to dataset and the reasons, if applicable for excluding any participant(s) from the dataset
- Method of PHO measurement including analysis software used
- Technique for measuring PHO – manual vs. semi-automated
    - the PHO measuring technique used such as ABC/2, largest diameter (where a manual method has been used)
    - Hounsfield unit threshold used for assessment of PHO (if applicable)

Participant level variables

Required

- Sex
- Age at presentation (years)
- Nature of symptom onset (awoke from sleep, last seen well, or awake at onset)
- Time from symptom onset to first scan (hours preferred or days, if hours not available)
- Glasgow Coma Scale (GCS) at presentation
- National Institutes of Health Stroke Scale (NIHSS) at presentation
- Single or multiple ICH (yes/no)
    - If participants with multiple ICH were included, how PHO was assessed
- Location of ICH – as per CHARTS location where lobar = frontal, parietal, temporal, occipital, insular regions; deep = basal ganglia, thalamus, internal capsule, external capsule, corpus callosum, and deep and periventricular white matter defined as white matter adjacent to or within approximately 10 mm of the lateral ventricular margin); infratentorial = (brainstem or cerebellum), uncertain = (probably lobar, probably deep, holohemispheric)
- ICH volume (mm^3 or ml) on first (diagnostic) scan
- PHO volume (mm^3 or ml) on first (diagnostic) scan
- Intraventricular extension on first (diagnostic) scan (yes/no)
- Time from symptom onset to second scan (hours preferred or days if hours not available)
- ICH volume (mm^3 or ml) on second scan
- PHO volume (mm^3 or ml) on second scan
- Time from symptom onset to third scan (hours preferred or days if hours not available)
- ICH volume (mm^3 or ml) on third scan
- PHO volume (mm^3 or ml) on third scan
- Type of functional outcome (ideally modified Rankin Scale score)
- Date of assessment of functional outcome
- Score on functional outcome
- Occurrence and date of death

Desirable

- Ethnicity
- First-ever or recurrent ICH
- Premorbid level of function; for example as measured by modified Rankin scale
- History of hypertension before ICH which led to inclusion in the study (yes/no/unknown)
- History of diabetes mellitus (yes/no/unknown)
- History of ischaemic stroke (yes/no/unknown)
- Whether on any oral or parenteral anticoagulant at ICH symptom onset (yes/no/unknown)
- Whether on antiplatelet therapy at ICH symptom onset (yes/no/unknown)
- Whether taking beta-blockers at ICH symptom onset (yes/no/unknown)
- Whether taking a statin at ICH symptom onset (yes/no/unknown)
- Whether taking immunosuppressive agents at ICH symptom onset -systemically administered steroids, steroid sparing agents (including Azathioprine, methotrexate, cyclosporin, tacrolimus, mycofenolate mofetil), disease modifying therapies for coexisting neurological disorders such as multiple sclerosis (yes/no/unknown)
- Pyrexia (defined as temperature >37.5 degrees Celsius) at presentation (yes/no)
- Systolic and diastolic blood pressure on admission (mmHg) or mean arterial blood pressure (mmHg) if systolic and diastolic blood pressure are unavailable
- Blood glucose (mmol/l or mg/dL), fibrinogen, haematocrit, platelet count (platelet number per litre) and plasma sodium (mmol/l) at presentation
- Whether an acute blood pressure lowering intervention (including – beta blocker, ACE-inhibitor, angiotensin 2 receptor blocker, calcium channel blocker, nitric oxide donor, alpha blocker, diuretic, centrally acting agent, other) was used (yes/no) following ICH
    - If an acute blood pressure lowering intervention was used, the timing of the intervention used in relation to symptom onset (<2 hours, 2–6hours, 6–48 hours and >48 hours after onset of ICH)
- Use of mannitol in first week after ICH onset (yes/no)
- Use of hypertonic saline in first week after ICH onset (yes/no)
- Presence of subarachnoid haemorrhage on first CT scan (yes/no)
- Any finger like projections of ICH on diagnostic scan
- Presence of CTA spot sign when CTA concurrently acquired with first CT scan (yes/no)
- Do-not-attempt resuscitation order instigated following ICH (yes/no)
    - If 'Yes', interval (days or hours) between ICH onset and do-not-attempt resuscitation order

Data management

1. Where data from an identified study are available within the Virtual International Stroke Trials Archive-ICH (VISTA-ICH http://www.virtualtrialsarchives.org/vista-ich; n=725), we will apply to obtain these data. We will also invite corresponding authors of eligible studies identified by the systematic review and not

already included in VISTA-ICH (including both observational studies and the control arms of randomised trials) to participate in the IPDMA and request anonymised datasets containing the study level variables and participant variables listed above.

2. We will use individual participant data from all collaborating studies, and consider, where possible using aggregate data from other eligible studies which do not contribute individual participant data.
3. We will require collaborating studies to supply a data dictionary with their data explaining the variables provided.
4. For each dataset, we will check the completeness, ranges, and values of the data provided. We will request the same dataset that has already been published. We will standardize the format and coding of the variables across the collaborating studies. Datasets obtained from collaborating studies will be combined to form a new master dataset, which will include a variable to indicate the original study. Data provided by VISTA will be identified as one dataset, because VISTA does not permit identification of the contributing studies.

   We will apply a consistent measure of PHO on the first, and any subsequent scans. Since previous studies have used different measures of PHO, we will request ICH volume and PHO volume on each participant, which will then permit calculation of the different measurements that have been used in previous studies, including absolute PHO volume (total lesion volume-ICH volume), relative PHO volume ([total lesion volume-ICH volume]/ICH volume), PHO expansion rate and oedema extension distance (which is the difference between the radius of a sphere equal to the total lesion volume and the radius of a sphere equal to ICH volume alone).[18] We will use absolute volume as our primary measure of PHO since this measure of PHO is the most commonly used measure of PHO..

Statistical analysis

1. **In adults who have a spontaneous ICH:**
   a. **Is the change in PHO volume between two selected time points in the first two weeks after ICH associated with longer term functional outcome (for example, three months after ICH onset)?**
   b. **Is the volume of PHO at a certain time point in the first two weeks after ICH associated with longer term functional outcome (for example, three months after ICH onset)?**

   We will perform a two-stage individual participant data meta-analysis of the association between PHO and outcome using logistic regression modelling. A random intercept and random coefficient for the study will be included in each model. We will explore each potential effect modifier of the association between PHO and outcome in turn, by examining the effect of PHO (measured either as a change over time (research question 1a) or at a certain time point (research question 1b); for example in the first 24 hours after ICH onset), on the likelihood of a poor outcome (e.g. modified Rankin scale 3-6) in a model. We will also consider examining the effect of PHO using an ordinal analysis of functional outcome through a proportional odds logistic regression. In all models polynomial terms will be considered to account for any associations which are non-linear on the logit scale. We will use these findings to inform development of a multivariable model to determine the association between PHO and outcome after accounting for clinical and imaging prognostic variables (such as GCS on

admission, participant age, ICH volume, ICH location (supratentorial vs. infratentorial). Such a model will quantify the relationship between PHO and outcome, and will identify those with ICH most likely to be adversely affected by PHO.

2. **How does the association between PHO and functional outcome vary**
    a. **according to the time points between which change in PHO volume is measured in the first two weeks after ICH onset?**
    b. **according to the time at which PHO volume is measured in the first two weeks after ICH onset?**

   We will assess whether the association between PHO and functional outcome varies according to PHO growth since ICH onset, and determine the time window of growth from ICH onset which is most closely associated with outcome. This will be established through graphical representation of the log-odds ratio for the association and its 95% confidence interval. We will look at the development of PHO over time by comparing PHO measurements at certain time points in relation to ICH onset – such as at 24 hours and 72 hours after ICH; but the specific time points explored will depend on data availability at each time point.
3. **Is the association between PHO volume and functional outcome, as outlined in 1a affected by:**
    c. **clinical variables such as participant age, blood pressure on admission?**
    d. **radiological variables such as ICH volume or ICH location?**
4. **Is the association between change in PHO volume and functional outcome, as outlined in question 1b affected by:**
    e. **clinical variables such as participant age, blood pressure on admission?**
    f. **radiological variables such as ICH volume or ICH location?**

   Where adequate data are available, heterogeneity in the association between PHO and functional outcome will be assessed in the subgroups specified above to determine whether these factors modify any association between PHO and outcome. Significant effect modifiers identified will be used to inform potential extensions to the multivariable models developed for research questions 1a and 1b. We will handle potential effect modifiers either singly or in groups based upon our knowledge of potential interactions between them; for example prior anticoagulant use increasing ICH volume size and PHO volume likely to be in turn affected by ICH volume. These analyses may lack sufficient statistical power so may be hypothesis generating only.

Risk of bias

We will assess bias risk (using established tools such as the Newcastle Ottawa scale for observational studies[19] and the Cochrane collaboration tool for trials http://methods.cochrane.org/bias/assessing-risk-bias- included-studies), for all included studies. We will compare studies which contribute data to this IPDMA with studies which do not and assess publication bias by visual inspection for funnel plot asymmetry (with and without studies where IPD is obtained). The Grades of Recommendation, Assessment, Development and Evaluation[20] will be used to evaluate the quality of the synthesised evidence.

Consistency of PHO measurement

To examine the consistency of the measurement of PHO across the different cohorts, a summary statistics table shall be presented for the ratio of ICH volume to PHO volume at a given timepoint (set of time points to be determined depending on data availability) after ICH onset.

Transfer of data and confidentiality

The preferred formats for data transfer are the following: Excel spreadsheet or a delimited text file, but we will accept other formats (Access database, SAS transport file, SPSS portable file, fixed-format text file). We will not collect participant-identifiable data. Authors are asked to de-identify data before transfer by removing all participant-identifiable data, and replacing them with a unique study ID number for each participant. All data sent will be held securely in the strictest confidence on password protected servers, and the final dataset will be held in a secure repository (DataShare https://datashare.is.ed.ac.uk/) which has achieved the 'Data Seal of Approval,' a peer-reviewed status as a digital trusted repository.

Publication policy

We intend to include and order the authors according to (a) their contribution to the design and execution of this study and (b) the number of eligible participants they contribute with a complete dataset. We will apply the ICMJE criteria for authorship. The VISTA-ICH collaboration will be listed as a group author name. We will share the results of our analyses with participating groups. All authors will have the opportunity to review the manuscript and approve the final version before submission to a journal.

References

1. Feigin VL et al. Worldwide stroke incidence and early case fatality reported in 56 population-based studies: a systematic review. Lancet Neurol 2009;8:355–69
2. Samarasekera N et al. Influence of intracerebral haemorrhage location on incidence, characteristics and outcome: population-based study. Stroke 2015;46:361-8
3. Ironside N et al. Perihematomal edema after spontaneous intracerebral haemorrhage. Stroke 2019;50:1626-1633. DOI: 10.1161/STROKEAHA.119.024965
4. The Hemorrhagic Stroke Academia Industry (HEADS) Roundtable participants Recommendations for Clinical Trials in ICH. Stroke 2020;51:1333-1338. DOI: 10.1161/STROKEAHA.119.027882
5. The Stroke Association State of play review – Haemorrhagic Stroke Research Priority Document 2014 https://www.stroke.org.uk/news/haemorrhagic-stroke-workshop-priority-setting
6. Levine JM et al. Early edema in warfarin-related intracerebral hemorrhage. Neurocrit Care 2007;7:58–63
7. Appelboom G et al. Volume-dependent effect of perihaematomal oedema on outcome for spontaneous intracerebral haemorrhages. J Neurol Neurosurg Psychiatry 2013;84:488–93
8. Ozdinc S et al. Prognostic value of perihematomal edema area at the initial ED presentation in patients with intracranial hematoma. Am J Emerg Med 2016;34:1241–6
9. Yang J et al. Prognostic Significance of Perihematomal Edema in Acute Intracerebral Hemorrhage. Stroke 2015;46:1009-13

10. Murthy SB et al. Rate of perihaematomal oedema expansion is associated with poor clinical outcomes in intracerebral haemorrhage. J Neurol Neurosurg Psychiatry 2016;87:1169–73.
11. Urday S et al. Rate of Perihematomal Edema Expansion Predicts Outcome After Intracerebral Hemorrhage. Crit Care Med 2016;44:790-797
12. Volbers B et al. Peak perihemorrhagic edema correlates with functional outcome in intracerebral hemorrhage. Neurology 2018;90:e1005–12
13. Grunwald Z et al. Perihematomal Edema Expansion Rates and Patient Outcomes in Deep and Lobar Intracerebral Hemorrhage. Neurocrit Care 2017;26:205–12
14. Selim M et al. Perihematoma edema: a potential translational target in intracerebral hemorrhage? Transl Stroke Res 2015;6:104-6
15. Wagner M et al. Sex differences in perihemorrhagic edema evolution after spontaneous intracerebral haemorrhage. Eur J Neurol 2012;19:1477-1481
16. Urday S et al. Targeting secondary injury in intracerebral haemorrhage – perihaematomal oedema. Nat Rev Neurol 2015;11:111-22
17. Volbers B et al. Semi-automatic volumetric assessment of perihemorrhagic edema with computed tomography. European J Neurol 2011;18:1323–8
18. Parry-Jones AR et al. Edema extension distance: outcome measure for phase II clinical trials targeting edema after intracerebral haemorrhage. Stroke 2015;46:e137-40
19. Wells GA, Shea B, O'Connell D, et al. The Newcastle-Ottawa Scale (NOS) for assessing the quality of nonrandomised studies in meta-analyses. The Ottawa Hospital Research Institute. http://www.ohri.ca/programs/clinical_epidemiology/oxford.asp
20. Iorio A, Spencer FA, Falavigna M, et al. Use of GRADE for assessment of evidence about prognosis: rating confidence in estimates of event rates in broad categories of patients. BMJ 2015;350:h870.

The association between peri-haematomal oedema and functional outcome after intracerebral haemorrhage - Individual participant data meta-analysis

Statistical Analysis Plan (not published elsewhere)

CONFIDENTIAL

**Version No**	1.0
**Date Finalised**	6th July 2022
**Author(s)**	Sharon Tuck; Prof. Chris Weir
**CI Name**	Dr. N Samarasekera
**CI Email address**	neshika.samarasekera@ed.ac.uk

Signatures	
**Trial Statistician: Prof. Chris Weir**	**Date:** **6 July 2022**
**Chief Investigator: Dr. N Samarasekera**	**Date:** **6.7.22**

**Document Control**		
**Version No**	**Date**	**Summary of Revisions**
1.0	06Jul2022	Initial Creation

Table of Contents

List of Abbreviations 28
1. Introduction 29
2. Statistical Methods section from the protocol 29
2.1 Study identification 29
2.2 Inclusion and exclusion criteria 29
2.3 Data collected 30
2.3 Data management 32
2.4 Risk of bias 32
3. Research questions 33
3.1 Primary research question 33
3.2 Secondary/additional research questions 33
4. Overall Statistical Principles 33
4.1 Missing data 34
5. List of Analyses 34
5.1 Descriptive statistics 34
5.2 Primary research question 35
5.2.1 Primary analysis 35
5.2.3 Sensitivity analysis 35
5.3 Secondary research questions 36
5.3.1 Secondary analysis 1 36
5.3.2 Secondary analysis 2 36
5.3.2.1 Analysis a – Change in PHO volume against estimated probability of poor functional outcome, by time of PHO measurement 36
5.3.2.2 Analysis b – PHO volume against estimated probability of poor functional outcome, by time of PHO measurement 37
5.3.3 Secondary analysis 3 37
5.3.3.1 Analysis c – Adjusted logistic regression of primary analysis (clinical) 37
5.3.3.2 Analysis d - Adjusted logistic regression of primary analysis (radiological) 38
5.3.3.3 Multivariable adjusted logistic regression model – primary analysis 38
5.3.4 Secondary analysis 4 39
5.3.4.1 Analysis e - Adjusted logistic regression of secondary analysis 1 (clinical) 39
5.3.4.2 Analysis f - Adjusted logistic regression of secondary analysis 1 (radiological) 39
5.3.4.3 Multivariable model – secondary analysis 1 39
5.3.5 Supporting analyses 40
6. Validation and QC 40
7. Data sharing 40

List of Abbreviations

Abbreviation	Full name
AE	Adverse event
AIC	Akaike Information Criterion
BIC	Bayesian Information Criterion
CHARTS	The Cerebral Haemorrhage Anatomical RaTing instrument
CT	Computed tomography
CTA	Computed tomography angiography
ECTU	Edinburgh Clinical Trials Unit
EED	Edema extension distance
GCS	Glasgow Coma Scale
ICH	Intracerebral haemorrhage
IPDMA	Individual participant data meta-analysis
ITT	Intention to treat
NIHSS	National Institutes of Health Stroke Scale
OED	Oedema extension distance
PHE	Peri-hematomal edema
PHO	Peri-haematomal oedema
RCT	Randomised controlled trial
SAE	Serious adverse event
SAP	Statistical analysis plan

1.Introduction

This document details the criteria to be used for the definition of the analysis populations and the statistical methodology for analysis for the association between peri-haematomal oedema (PHO) and functional outcome after intracerebral haemorrhage (ICH).

Since differences between studies preclude standard meta-analysis based on aggregated data, we intend to use an individual participant data meta-analysis (IPDMA) to firstly explore the strength of the association between PHO and outcome after ICH and secondly to identify variables which may modify the strength of the association of PHO with outcome.

This statistical analysis plan (SAP) applies to this IPDMA, which is restricted to completed RCTs and observational studies primarily assessing the association between PHO and outcome after ICH. This SAP has been compiled according to the Edinburgh Clinical Trials Unit (ECTU) standard operating procedure (SOP) 'Statistical Analysis Plans v6.0' and has been written based on information contained in the study protocol version 1.1, dated 15th June 2022 and the PROSPERO version of the protocol [1]"

2.Statistical Methods section from the protocol

2.1Study identification

On 4 April 2021 we completed a comprehensive search of electronic databases (Embase (1980-) and Medline (1950-); appendix 1), hand searched bibliographies of relevant studies, and performed forward citation searching for each included article by using Google Scholar to identify relevant studies (registered on PROSPERO CRD42020157088). We excluded conference abstracts.

2.2Inclusion and exclusion criteria

Study level inclusion criteria:

- Acute spontaneous (non-traumatic) supratentorial or infratentorial ICH confirmed by CT brain imaging.
- Repeat CT brain imaging performed at least once up to 14 days after the first brain imaging study.
- Observational cohort studies or control arms of randomised trials that measured PHO and functional outcome (using modified Rankin scale or similar measure) after ICH.
- Randomised trials which measured PHO and functional outcome (using modified Rankin scale or similar measure) after ICH, where the intervention used should not affect PHO; or the control arm of randomised trials only if the intervention may have affected PHO (for example, steroids, mannitol, hypertonic saline).

Exclusion criteria:
Study level:
- Studies reporting participants who are included in other publications that reported a larger cohort.
- Studies solely using MRI to assess PHO (since CT is the most frequently used imaging modality for participants with ICH and is reliable for the semiautomated assessment of PHO)
- Studies involving <5 participants with spontaneous ICH

Participant level:
- Age less than 18 years.
- Secondary causes of ICH (including trauma, tumour, intracranial aneurysm, arteriovenous malformation, arteriovenous fistula, cavernous malformation, venous thrombosis, moyamoya syndrome, reversible cerebral vasoconstriction syndrome, or haemorrhagic transformation of ischaemic stroke).
- Exclusively intraventricular/subarachnoid/subdural/extradural haemorrhage.
- Participants treated with surgery, mannitol or steroids following their ICH (since these might also affect PHO)
- Participants where first (diagnostic) CT was performed more than 72 hours after ICH onset or where the time from ICH onset to first CT was unknown.
- Participants who might be eligible for inclusion in the IPDMA but whose data were not originally published in the specific study.

2.3Data collected

Study level variables:
- *first author of study and date*
- *study period (years)*
- *country*
- *funding source*
- *participants identified prospectively vs. retrospectively*
- *inclusion and exclusion criteria*
- *prespecified CT scanning protocol used*
- *prospective outcome ascertainment used*
- *time point of measuring functional outcome and method (e.g. modified Rankin scale)*
- *number of participants provided by each study to dataset and reasons, if applicable, for excluding any participants from the dataset*
- *method of PHO measurement including analysis software used*
- *technique for measuring PHO (manual vs. semi-automated)*
    - *the PHO measuring technique used such as ABC/2, largest diameter (where a manual method has been used)*
    - *Hounsfield unit threshold used for assessment of PHO (if applicable)*

Participant-level variables
Required
- *Sex*
- *Age at presentation (year)*
- *Nature of symptom onset (awoke from sleep; last seen well; awake at onset)*
- *Time from symptom onset to first scan (hour preferred, but days if hours not available)*
- *Glasgow Coma Scale (GCS) at presentation*

- *National Institutes of Health Stroke Scale (NIHSS) at presentation*
- *Single or multiple ICH (yes/no)*
    - *If participants with multiple ICH were included, how PHO was assessed*
- *Location of ICH – as per CHARTS location where lobar = frontal, parietal, temporal, occipital, insular regions; deep = basal ganglia, thalamus, internal capsule, external capsule, corpus callosum, and deep and periventricular white matter defined as white matter adjacent to or within approximately 10 mm of the lateral ventricular margin); infratentorial = (brainstem or cerebellum), uncertain = (probably lobar, probably deep, holohemispheric)*
- *ICH volume on first diagnostic scan (mm^3 or ml)*
- *PHO volume of first diagnostic scan (mm^3 or ml)*
- *Intraventricular extension on first diagnostic scan (yes/no)*
- *Time from symptom onset to second scan (hours preferred or days if hours not available)*
- *ICH volume on second scan (mm^3 or ml)*
- *PHO volume on second scan (mm^3 or ml)*
- *Time from symptom onset to third scan (hours preferred or days if hours not available)*
- *ICH volume on third scan (mm^3 or ml)*
- *PHO volume on third scan (mm^3 or ml)*
- *Type of functional outcome (ideally modified Rankin scale)*
- *Date of assessment of functional outcome*
- *Score on functional outcome*
- *Occurrence and date of death*

Desirable

- *Ethnicity*
- *First-ever or recurrent ICH*
- *Premorbid level of function (e.g. measured by modified Rankin scale)*
- *History of hypertension before ICH which led to inclusion in the study (yes/no/unknown)*
- *History of diabetes mellitus (yes/no/unknown)*
- *History of ischaemic stroke (yes/no/unknown)*
- *Whether on any oral or parenteral anticoagulant at ICH symptom onset (yes/no/unknown)*
- *Whether on antiplatelet therapy at ICH symptom onset (yes/no/unknown)*
- *Whether taking beta-blockers at ICH symptom onset (yes/no/unknown)*
- *Whether taking a statin at ICH symptom onset (yes/no/unknown)*
- *Whether taking immunosuppressive agents at ICH symptom onset -systemically administered steroids, steroid sparing agents (including Azathioprine, methotrexate, cyclosporin, tacrolimus, mycofenolate mofetil), disease modifying therapies for coexisting neurological disorders such as multiple sclerosis (yes/no/unknown)*
- *Pyrexia (defined as temperature >37.5 degrees Celsius) at presentation (yes/no)*
- *Systolic and diastolic blood pressure on admission (mmHg) or mean arterial blood pressure (mmHg) if systolic and diastolic blood pressure are unavailable*
- *Blood glucose (mmol/l or mg/dL), fibrinogen, haematocrit, platelet count (platelet number per litre) and plasma sodium (mmol/l) at presentation*
- *Whether an acute blood pressure lowering intervention (including – beta blocker, ACE-inhibitor, angiotensin 2 receptor blocker, calcium channel blocker, nitric oxide donor, alpha blocker, diuretic, centrally acting agent, other) was used (yes/no) following ICH*
- *If an acute blood pressure lowering intervention was used, the timing of the intervention used in relation to symptom onset (<2 hours, 2–6hours, 6–48 hours and >48 hours after onset of ICH)*
- *Use of mannitol in first week after ICH onset (yes/no)*

- *Use of hypertonic saline in first week after ICH onset (yes/no)*
- *Presence of subarachnoid haemorrhage on first CT scan (yes/no)*
- *Any finger like projections of ICH on diagnostic scan*
- *Presence of CTA spot sign when CTA concurrently acquired with first CT scan (yes/no)*
- *Do-not-attempt resuscitation order instigated following ICH (yes/no)*
    - *If 'Yes', interval (days or hours) between ICH onset and do-not-attempt resuscitation order*

2.3 Data management

5. *Where data from an identified study are available within the Virtual International Stroke Trials Archive-ICH, we will apply to obtain these data. We will also invite corresponding authors of eligible studies identified by the systematic review and not already included in VISTA-ICH (including both observational studies and the control arms of randomised trials) to participate in the IPDMA and request anonymised datasets containing the study level variables and participant variables listed above.*
6. *We will use individual participant data from all collaborating studies, and consider, where possible using aggregate data from other eligible studies which do not contribute individual participant data.*
7. *We will require collaborating studies to supply a data dictionary with their data explaining the variables provided.*
8. *For each dataset, we will check the completeness, ranges, and values of the data provided. We will request the same dataset that has already been published. We will standardize the format and coding of the variables across the collaborating studies. Datasets obtained from collaborating studies will be combined to form a new master dataset, which will include a variable to indicate the original study. Data provided by VISTA will be identified as one dataset, because VISTA does not permit identification of the contributing studies.*

   We will apply a consistent measure of PHO on the first, and any subsequent scans. Since previous studies have used different measures of PHO, we will request ICH volume and PHO volume on each participant, which will then permit calculation of the different measurements that have been used in previous studies, including absolute PHO volume (total lesion volume-ICH volume), relative PHO volume ([total lesion volume-ICH volume]/ICH volume), PHO expansion rate and oedema extension distance (which is the difference between the radius of a sphere equal to the total lesion volume and the radius of a sphere equal to ICH volume alone). We will use absolute volume as our primary measure of PHO since this measure of PHO is the most commonly used measure of PHO.

2.4Risk of bias

We will assess bias risk (using established tools such as the Newcastle Ottawa scale for observational studies and the Cochrane collaboration tool for trials http://methods.cochrane.org/bias/assessing-risk-bias- included-studies), for all included studies. We will compare studies which contribute data to this IPDMA with studies which do not and assess publication bias by visual inspection for funnel plot asymmetry (with and without studies where IPD is obtained). The Grades of Recommendation, Assessment, Development and Evaluation[20] will be used to evaluate the quality of the synthesised evidence.

3. Research questions

3.1Primary research question

Is change in absolute PHO volume between two selected time points in the first two weeks after ICH associated with longer term functional outcome (for example, three months after ICH onset?

3.2Secondary/additional research questions

1. Is volume of PHO measured at a certain time point in the first two weeks after ICH associated with longer term functional outcome (for example, three months after ICH onset)?

2. Does the association between PHO and functional outcome vary:
   a. according to the time points between which change in PHO volume is measured in the first two weeks after ICH onset?
   b. according to the time at which PHO volume is measured in the first two weeks after ICH onset?

3. Is the association between PHO volume and functional outcome, as outlined in secondary research question 1, affected by:
   c. clinical variables such as participant age, blood pressure on admission?
   d. radiological variables such as ICH volume, ICH location?

4. Is the association between change in PHO volume and functional outcome, as outlined in the primary research question, affected by:
   e. clinical variables such as participant age, blood pressure on admission?
   f. radiological variables such as ICH volume, ICH location?

4.Overall Statistical Principles

In general terms, categorical data will be presented using counts and percentages, whilst continuous variables will be presented – according to their distribution – using the mean, median, standard deviation (SD), minimum, maximum, lower (Q1) and upper (Q3) quartiles and number of patients with an observation (n).

Distributional assumptions underlying the statistical analyses will be assessed by visual inspection of residual plots. Normality will be examined by normal probability plots. If the distributional assumptions for the parametric approach are not satisfied, further data transformation (to alleviate substantial skewness (i.e.log-transformation) or to stabilise the variance), or other suitable methods will be considered. This will be documented in the statistical results report together with the reasoning supporting the action taken, if applicable.

ST004-SAP Template /v4.0/25 Mar 2021

All analyses and data manipulations will be carried out using RStudio R-3.6.1 (from the University of Edinburgh's Software Centre).

4.1Missing data

Where there is missing datum for a variable, in the first instance, those records will be removed from any formal statistical analysis relating to that variable, unless otherwise specified. In tabulations, numbers of missing observations will be provided, but percentages will not include them.

5.List of Analyses

5.1Descriptive statistics

A descriptive summary of all required and desirable variables (specified in section 2.3) shall be presented separately by:

- Trial
- Absolute PHO volume in the first two weeks after ICH onset (i.e. if PHO volume is '0.0' then no PHO present, binary no vs. yes) and overall
- Functional outcome, defined by modified Rankin score (i.e. 0-2 good outcome vs. 3-6 poor outcome)

The distribution of absolute PHO volume shall be illustrated showing how PHO volume varies according to time from ICH onset for each study separately and the whole study cohort; subdivided by ICH volume (<30ml vs. ≥30ml).

In addition to this, a series of line graphs shall be created to visually illustrate the following data:

- Time PHO volume measured along the x-axis
- PHO volume along the y-axis

For each study separately, two line graphs shall be presented for participants with poor functional outcome and for participants with 'good' functional outcome according to the modified Rankin score.

5.2Primary research question

5.2.1Primary analysis

In adults who have a spontaneous ICH, is the change in absolute PHO volume in the first two weeks after ICH associated with longer term functional outcome (three months after ICH onset)?

Absolute PHO volume is recorded in the database as '*ScanX*_PHOvolume'; where X is either 1, 2 or 3.

The R statistical package shall be used to perform the statistical analysis and a two-stage approach shall be used to obtain the model estimates. The two-stage approach is preferred here as the studies included are large and this enables use of restricted maximum likelihood estimation (REML; reducing the downward bias in between-study variance estimates) which is not available under the one-stage approach. Random-effects modelling will allow the true association to be different for each study, allowing for heterogeneity between studies.

A two-stage, unadjusted, random-effects logistic regression meta-analysis shall be performed with REML estimation followed by the Hartung-Knapp adjustment, which controls for the uncertainty in between-study variance estimates by inflating the width of the confidence intervals [3].

1. To generate the aggregate data, a logistic regression shall be performed for each study to obtain log odds ratio estimates and their standard errors from the following:
    - Poor functional outcome at 90 days post ICH onset (modified Rankin scale: 0-2 good vs. 3-6 poor) – dependent
    - Change in absolute PHO volume between baseline measurement (1st scan) and 72 hour measurement[1] in the first two weeks after ICH (continuous)– independent

2. Apply a random-effects meta-analysis (i.e. `metagen`) to the combined aggregate data using REML (`method.tau = "REML"`) estimation with a Hartung-Knapp adjustment (hakn=TRUE) for random effects modelling to obtain a summary estimate, 95% CI for average effect and the between study heterogeneity (i.e. τ^2).

3. If the pre-specified methods do not work (or assumptions aren't satisfied), then alternative methods shall be investigated.

4. A forest plot for the above model shall be produced using the random effects meta-analysis with REML estimation along with odds ratios for each study and the overall pooled odds ratio and 95% confidence intervals. The value 'I^2' shall also be reported to show whether there is any observed between-study heterogeneity.

5.2.3Sensitivity analysis

To examine the sensitivity of the primary analysis the random effects model shall be compared with the common effects (fixed-effect) model which assumes that the true association is the same in each study. A forest plot of the common effects model shall also be presented.

[1] If not much data available for the 72 hour timepoint then the 24 hour timepoint shall be used.

ST004-SAP Template /v4.0/25 Mar 2021

5.3Secondary research questions

5.3.1 Secondary analysis 1

An analysis following the same procedure as the primary outcome shall be performed to examine whether the volume of PHO measured at a certain time point in the first two weeks after ICH is associated with longer term functional outcome.

To generate the aggregate data, the following variables shall be entered into a logistic regression model for each study:

- Poor functional outcome at 90 days post ICH onset (modified Rankin scale: 0-2 good vs. 3-6 poor – dependent
- Volume of PHO at a certain time point (i.e. either 24 or 72 hours - the time point chosen will be one with the greatest quantity of data) in the first two weeks after ICH (continuous) - independent

Once all aggregate data have been collected for this model, follow the same instructions as defined above for the primary analysis.

5.3.2 Secondary analysis 2

5.3.2.1 Analysis a – Change in PHO volume against estimated probability of poor functional outcome, by time of PHO measurement

To examine whether the association between PHO volume and functional outcome vary according to the time points between which changes in PHO volume is measured in the first two weeks after ICH onset, a series of line graphs shall be produced showing the change in PHO volume between pre-specified time points (x-axis) against the estimated probability of poor functional outcome (y-axis).

To generate the aggregate data, the following variables will be entered into a logistic regression model for each study:

- Poor functional outcome at 90 days post ICH onset (modified Rankin scale: 0-2 good vs. 3-6 poor – dependent
- Change in PHO volume between certain time points (see below) – independent

Once all aggregate data have been collected for the model, a fitted logistic curve for each study shall be plotted showing the estimated probability of poor functional outcome against change in PHO volume (for those particular time points). The binary responses to functional outcome (i.e. poor or good according to the modified Rankin score) shall also be plotted at the top and bottom of the y-axis scale for each study.

This process shall be carried out for each of the following time points separately, resulting in a separate graph for each set of changes between time points (*subject to change depending on the data available at the time of analysis*):

- Change between 24 hours after baseline and baseline
- Change between 72 hours after baseline and baseline
- Change between 1 week after baseline and baseline

5.3.2.2 Analysis b – PHO volume against estimated probability of poor functional outcome, by time of PHO measurement

To examine whether the association between PHO volume and functional outcome varies according to the time at which PHO volume is measured in the first two weeks after ICH onset, a similar analysis to '*Analysis a*' above shall be performed. A series of fitted logistic curve graphs for each study shall be produced showing PHO volume (x-axis) against the estimated probability of poor functional outcome (y-axis).

This analysis shall be carried out for each time point of PHO measurement separately, resulting in a separate graph for each time point. Please note that the final list of time points will be determined depending on the data available at the time of analysis:

- Baseline
- 24 hours after baseline
- 72 hours after baseline
- 1 week after baseline

5.3.3 Secondary analysis 3

5.3.3.1 Analysis c – Adjusted logistic regression of secondary analysis 1 (clinical)

To examine if the association between volume of PHO measured at either 24 or 72 hours and poor functional outcome is affected by clinical variables, a two-stage, random-effects logistic regression meta-analysis (with REML estimation and a Hartung-Knapp adjustment) shall be performed adjusting for each individual clinical variable of interest separately.

To generate the aggregate data, an adjusted logistic regression shall be performed for each study to obtain log odds ratio estimates and their standard errors from the following:

- Poor functional outcome (yes/no) – dependent
- PHO volume at either 24 or 72 hours in the first two weeks after ICH (continuous) - independent
- Clinical variable of interest (continuous/categorical/binary) – independent
- Interaction between PHO volume and clinical variable of interest[2]

Once the aggregate data has been collected, follow the same instructions as defined in section 5.3.1 for the primary analysis. A table presenting the frequencies of outcome and numbers of participants in each stratum of the clinical variable of interest, model coefficient, standard error, p-value, odds ratio and 95% confidence intervals from each clinical variable shall be presented.

The set of clinical variables of interest for our adjusted model are:

1. Age at presentation (years) – continuous[3]
2. Gender
3. Presence of intraventricular extension of ICH – binary; yes/no

[2] An interaction term will only be considered if there is any evidence that such inclusion would be appropriate based on prior analysis and shall be based on magnitude of effects, rather than statistical significance.

[3] Linearity shall be checked, and the relationships between continuous variables and outcome will be check to see whether linearity is held. If variable is non-linear then fractional polynomials shall be used.

ST004-SAP Template /v4.0/25 Mar 2021

4. GCS on admission – binary; yes/no
5. History of hypertension – binary; yes/no
6. Systolic BP on admission
7. Use BP lowering (only for MISTIE, Castro, Helsinki and NICHE studies; include only in univariate analysis)
8. Do not resuscitate – binary; yes/no

5.3.3.2 Analysis d - Adjusted logistic regression of secondary analysis 1 (radiological)

The similar analysis to that described in 'Analysis c' shall be carried out using the following radiological variables (instead of the clinical variables):

1. ICH volume – continuous
2. ICH location – categorical; infratentorial vs. lobar vs. non-lobar
3. Time from ICH onset to baseline imaging - continuous

5.3.3.3 Multivariable adjusted logistic regression model – secondary analysis 1

Based on the results from '*Analysis c*' and '*Analysis d*', a multivariable logistic regression model shall be developed using the clinical and radiological variables of interest. We will use statistical and clinical judgement on whether a variable of interest should be included in the final multivariable model. Where there is clinical evidence that one of our clinical or radiological variables is known to be associated with poor functional outcome then this variable will be included in the model regardless of whether or not there is evidence of statistical significance. Two-way interactions shall also be considered in the model. The model specified in section 5.3.1 will be used, with additional adjustment for the clinical and radiological variables selected above.

A table presenting the overall pooled odds ratio along with odds ratios for each study and 95% confidence intervals shall be presented along with the C-statistic (i.e. area under the ROC curve) to examine discrimination and the Brier score to examine calibration across models. The AIC and BIC statistics shall also be examined to judge and compare the fit of each model.

The final model shall also be internally validated by Bootstrap validation which by resampling from the full original data set allows all of the available data to contribute to model development [3]. In addition, bootstrapping allows optimism-adjusted values of the C-statistic and Brier score to be calculated.

5.3.4 Secondary analysis 4

5.3.4.1 Analysis e - Adjusted logistic regression of primary analysis (clinical)

To examine if the association between the change in PHO volume (between baseline and 72 hours) and poor functional outcome is affected by clinical variables, a two-stage, random-effects logistic regression meta-analysis (with REML estimation and a Hartung-Knapp adjustment) shall be performed adjusting for each individual clinical variable of interest separately.

To generate the aggregate data, an adjusted logistic regression shall be performed for each study to obtain log odds ratio estimates and their standard errors from the following:

- Poor functional outcome at 90 days post ICH onset (modified Rankin scale: 0-2 good vs. 3-6 poor – dependent
- Change in PHO volume between baseline measurement (1st scan) and 72 hours – independent
- Clinical variable of interest (continuous/categorical/binary) – independent
- Interaction between change in PHO volume and clinical variable of interest[4]

Once the aggregate data has been collected, follow the same instructions as defined above in Secondary outcome 3, Analysis c (Section 5.3.3.1) using the same pre-specified set of clinical variables.

5.3.4.2 Analysis f - Adjusted logistic regression of primary analysis (radiological)

An equivalent analysis to '*Analysis d*' (Section 5.3.3.2) will be performed to examine if the association between the change in PHO volume and poor functional outcome is affected by radiological variables.

5.3.4.3 Multivariable model – primary analysis

Based on the results from '*Analysis e*' and '*Analysis f*', a multivariable logistic regression model shall be developed using the clinical and radiological variables of interest. We will use statistical and clinical judgement on whether a variable of interest should be included in the final multivariable model. Where there is clinical evidence that one of our clinical or radiological variables is known to be associated with poor functional outcome then this variable will be included in the model regardless of whether or not there is evidence of statistical significance. Interactions between the change in PHO volume and included clinical and radiological variables shall also be included in the final models. The model specified in section 5.3.1 will be used, with additional adjustment for the clinical and radiological variables selected above.

A table presenting the pooled odds ratio and 95% confidence intervals for each multivariable model shall be presented along with the C-statistic (i.e. area under the ROC curve) to examine discrimination and the Brier score to examine calibration across models. The AIC and BIC statistics shall also be examined to judge and compare the fit of each model. Bootstrap validation will also be applied as in section 5.3.3.3.

[4] An interaction term will only be considered if there is any evidence that such inclusion would be appropriate based on prior analysis and shall be based on magnitude of effects, rather than statistical significance.

ST004-SAP Template /v4.0/25 Mar 2021

5.3.5 Supporting analyses

1. To examine the consistency of the measurement of PHO across the different cohorts, a summary statistics table shall be presented for the ratio of ICH volume to PHO volume at a given timepoint (set of time points to be determined depending on data availability) after ICH onset.

2. As a further supporting analysis, subject to data availability, the absolute ICH volume shall be analysed as a secondary measure of PHO, since PHO is likely to be closely related to mass effected generated by an ICH (i.e. a predictor of outcome). A similar model to that used in the primary analysis shall be performed but using the absolute volume of ICH as the predictor of outcome.

6. Validation and QC

A second statistician will perform separate programming and checking of primary outcome results and conclusions. The statistical report will also be read and sense-checked.

7. Data sharing

The data used for this project are governed by a series of data sharing agreements with the source data providers, and are therefore not the property of the University of Edinburgh. Requests for data sharing will need to be directed towards the source data providers.

8. References

[1] "IPDMA PROSPERO protocol_CRD42021253263.pdf" Z:\ECTU Current Trials\1 CURRENT PROJECTS\ICH PHO IPDMA\TMF\1 STUDY DOCS\1 CURRENT\Protocol\Current\IPDMA PROSPERO protocol_CRD42021253263.pdf.

[2] G. Wells, B. Shea , D. O'Connell and et al, "The Newcastle-Ottawa Scale (NOS) for assessing the quality of nonrandomised studies in meta-analyses.," [Online]. Available: http://www.ohri.ca/programs/clinical_epidemiology/oxford.asp.

[3] J. H. &. G. Knapp, *STATISTICS IN MEDICINE,* vol. 20, pp. 3875-3889, 2001.

[4] E. W. Steyerberg, "Clinical Prediction Models," in *A Practical Approach to Development, Validation and Updating*, Springer, 2009, pp. 299 - 304.

[5] Adrian R. Parry-Jones, Xia Wang, Shoichiro Sato, W.Andrew Mould, Andy Vail, Craig S. Anderson and Daniel F. Hanley, "Edema Extension Distance: Outcome Measure for Phase II Clinical Trials Targeting Edema After Intracerebral Hemorrhage," Vols. Volume 46, Issue 6,, no. https://doi.org/10.1161/STROKEAHA.115.008818, pp. e137-e140, June 2015.
